# Stage 1 type 1 diabetes memory B lymphocytes transcriptionally differ from healthy controls and harbor insulin-binding specificities

**DOI:** 10.1093/immhor/vlaf053

**Published:** 2025-11-19

**Authors:** Lindsay E Bass, Wyatt J McDonnell, Christina T Brannon, Nilesh P Kumar, Simon A Mallal, Ivelin S Georgiev, James W Thomas, Daniel J Moore, Rachel H Bonami

**Affiliations:** Department of Pathology, Microbiology and Immunology, Vanderbilt University Medical Center (VUMC), Nashville, TN 37232, United States; Vanderbilt University, Nashville, TN 37232, United States; Department of Pathology, Microbiology and Immunology, Vanderbilt University Medical Center (VUMC), Nashville, TN 37232, United States; Vanderbilt University, Nashville, TN 37232, United States; Department of Medicine, Division of Rheumatology and Immunology, VUMC, Nashville, TN 37232, United States; Vanderbilt University, Nashville, TN 37232, United States; Department of Pathology, Microbiology and Immunology, Vanderbilt University Medical Center (VUMC), Nashville, TN 37232, United States; Vanderbilt University, Nashville, TN 37232, United States; Department of Medicine, Division of Infectious Diseases, VUMC, Nashville, TN 37232, United States; Institute for Immunology and Infectious Diseases, Murdoch University, Perth, Western Australia 6150, Australia; Department of Pathology, Microbiology and Immunology, Vanderbilt University Medical Center (VUMC), Nashville, TN 37232, United States; Vanderbilt University, Nashville, TN 37232, United States; Vanderbilt Center for Antibody Therapeutics, VUMC, Nashville, TN 37232, United States; Department of Pathology, Microbiology and Immunology, Vanderbilt University Medical Center (VUMC), Nashville, TN 37232, United States; Vanderbilt University, Nashville, TN 37232, United States; Department of Medicine, Division of Rheumatology and Immunology, VUMC, Nashville, TN 37232, United States; Vanderbilt Center for Immunobiology, Nashville, TN 37232, United States; Vanderbilt Institute for Infection, Immunology, and Inflammation, Nashville, TN 37232, United States; Department of Pathology, Microbiology and Immunology, Vanderbilt University Medical Center (VUMC), Nashville, TN 37232, United States; Vanderbilt University, Nashville, TN 37232, United States; Vanderbilt Center for Immunobiology, Nashville, TN 37232, United States; Vanderbilt Institute for Infection, Immunology, and Inflammation, Nashville, TN 37232, United States; Department of Pediatrics, VUMC, Nashville, TN 37232, United States; Department of Pathology, Microbiology and Immunology, Vanderbilt University Medical Center (VUMC), Nashville, TN 37232, United States; Vanderbilt University, Nashville, TN 37232, United States; Department of Medicine, Division of Rheumatology and Immunology, VUMC, Nashville, TN 37232, United States; Vanderbilt Center for Immunobiology, Nashville, TN 37232, United States; Vanderbilt Institute for Infection, Immunology, and Inflammation, Nashville, TN 37232, United States

**Keywords:** autoimmunity, diabetes, antibodies, B cells, single-cell sequencing

## Abstract

Autoreactive B cell activity defines the earliest detectable stage (Stage 1) of type 1 diabetes (T1D) but is incompletely understood, particularly for B cells reactive against the key T1D autoantigen, insulin. To test whether Stage 1 T1D B cells are transcriptionally rewired compared to healthy individuals, we performed single-cell transcriptional, phenotypic, and immune repertoire profiling of CD19^+^ cells isolated from the peripheral blood of Stage 1 T1D individuals, identified via Type 1 Diabetes TrialNet as being positive for ≥ 2/5 islet autoantibodies, and healthy controls. Stage 1 T1D memory B cells upregulated *n* = 122 genes compared to healthy controls, including genes involved in actin cytoskeleton rearrangement, B cell receptor (BCR) signaling, and antigen presentation, and exhibited reduced BCR somatic hypermutation, particularly in atypical-like memory B cells. Clonally expanded B cells in the atypical-like memory subset of Stage 1 T1D individuals exhibited avidity driven insulin-binding specificities, without polyreactivity to HEp-2 cell autoantigens. Insulin-binding B cells showed non-significant upregulation of genes involved in key B cell functions. Our findings highlight transcriptional and BCR-repertoire differences in Stage 1 T1D B cells with potential for optimization as future screening tools to identify rare, autoreactive B cells and biomarkers of T1D progression.

## Introduction

B cells have been implicated in human type 1 diabetes (T1D) pathogenesis and can differentiate to produce islet autoantibodies used to inform risk and diagnosis of T1D.^1^^,^^2^ In humans, increased B cell infiltration of islets corresponds with more aggressive and earlier disease onset.^3^ B cell depletion through a single dose of rituximab temporarily preserved beta cell function in new-onset T1D individuals.^4^^,^^5^ Studies in the non-obese diabetic (NOD) mouse model of T1D suggest that B cells contribute to disease through autoantigen presentation to autoreactive T cells.^6–11^ Disruption of B cell/T cell interaction through abatacept treatment (CTLA4-Ig) delayed beta cell decline in new-onset T1D individuals,^12^ while non-responders showed increased B cell frequencies and elevated B cell genes, supporting a role for B cell help in human T1D.^13^

A single nucleotide polymorphism (SNP) in the phosphatase *PTPN22*, which impairs B cell receptor (BCR) signaling, is associated with T1D and other autoimmune diseases.^14^ Studies of this polymorphism in a mouse model showed that it enhanced selection of autoreactive B cell specificities due to faulty immune tolerance checkpoints.^15^ These studies provide an example of how B cell reprogramming can influence T1D risk and led us to hypothesize that transcriptional differences exist amongst specific B cell subsets in individuals at the earliest stage of detectable T1D (Stage 1) compared to healthy controls. In support of this hypothesis, a recent report used LInking B cell Receptor to Antigen specificity through SEQuencing (LIBRA-seq)^16^ to identify islet antigen-reactive B cells and profile gene expression changes in antigen-reactive cells in autoantibody positive pre-diabetic and recent onset T1D.^17^ Autoantibody positive pre-diabetic individuals (2+ islet autoantibody positive) can be further classified into disease stages, as follows: Stage 1 (normal oral glucose tolerance test [OGTT] results) and Stage 2 (impaired OGTT results).^1^^,^^2^ This study did not distinguish between Stage 1 and Stage 2 T1D and thus did not eliminate potential confounding effects that dysregulated blood glucose may have on gene expression profiles observed in B cells. Therefore, we sought to analyze the gene expression and phenotype of CD19^+^ B cells from the peripheral blood of Stage 1 T1D individuals exclusively, compared to non-diabetic, first-degree relative controls.

Insulin recognition by both B and T cells is required for spontaneous T1D in NOD mice.^6^^,^^18^^,^^19^ Consistent with a key role for insulin-binding B cells in T1D, therapies that selectively deplete anti-insulin B cells prevent diabetes in NOD mice.^20^^,^^21^ Insulin autoantibody titers correlate with disease progression in humans,^22^^,^^23^ but insulin autoantibodies are often transient and wane by diabetes onset.^24^^,^^25^ This highlights the ever-changing immune landscape of T1D and supports the need to study each distinct disease stage independently. Only a small number of insulin-binding BCRs have been characterized in new onset (Stage 3)/late stage (Stage 4) T1D individuals.^17^^,^^26–29^ Additionally, exogenous insulin therapy to treat T1D drives “foreign” immune responses,^30^ emphasizing the need to study the development of “true” insulin autoimmunity during the pre-diabetes interval. Few studies to date have examined insulin-binding B cells in pre-diabetic T1D individuals.^17^^,^^31^ In these studies, the precise T1D stage (1 vs 2) of the donors was not noted, and only a few BCR sequences were published which showed demonstrable insulin-binding (at < 10 µg/ml mAb concentrations).^17^^,^^31^ An unbiased immune repertoire study of immunoglobulin H (IgH) sequences identified in pancreatic islets, pancreatic and “irrelevant” lymph nodes, spleen, and peripheral blood from individuals with established T1D reported overabundance of some VH gene segments, but did not allow for BCR antigen specificity to be determined, as light chains were not sequenced.^32^ We thus set out to identify new methods, using B cell gene expression and BCR repertoire, to identify insulin-binding B cells in an unselected B cell repertoire exclusively in Stage 1 T1D individuals.

Here, we identify differentially expressed genes within specific B cell subsets that are increased in Stage 1 T1D individuals, relative to non-diabetic, first-degree relative controls, including genes involved in BCR-mediated actin rearrangement, signaling, and antigen presentation. We additionally find that clonally expanded memory B cells in Stage 1 T1D individuals, which upregulate atypical B cell genes and exhibit low CD21 surface expression, contain insulin-binding specificities that are not polyreactive with HEp-2 cell antigens. B cells expressing ELISA-validated insulin-binding BCRs showed upregulation of several genes involved in key B cell functions relative to clonally expanded, non-insulin-binding B cells, as well as non-clonally expanded T1D B cells from the same cluster, albeit in a non-statistically significant manner given the expected rarity of insulin-binding B cell clones captured per individual. These findings highlight the utility of transcriptional and BCR repertoire profiling methods to identify rare, insulin binding B cells to better characterize these pathogenic cells.

## Materials and methods

### Sex as a biological variable

Male and female individuals were represented in both groups in this study (56% female in Stage 1 T1D and 63% female in healthy controls, Table 1). This study was underpowered to formally examine sex as a biological variable, but participant-level sex is included as metadata.

**Table 1.** Participant demographics.

Participant ID	Group**^a^**	Age	Sex	Positive islet autoantibodies**^b^^,^^c^^,^^f^**	Glucose tolerance test results**^e^^,^^f^**	T1D Stage
1	Stage 1 T1D	29	F	GAD65, ICA, ZNT8	Normal	1
2	Stage 1 T1D	17	F	GAD65**^d^**	Normal	1
3	Stage 1 T1D	12	M	MIAA, GAD65, IA2, ZNT8	Normal	1
4	Stage 1 T1D	19	F	GAD65, IA2, ICA, ZNT8	Normal	1
5	Stage 1 T1D	21	M	GAD65, ZNT8	Normal	1
6	Stage 1 T1D	18	M	MIAA, GAD65, ZNT8	Normal	1
7	Stage 1 T1D	8	M	GAD65, IA2, ICA, ZNT8	Normal	1
8	Stage 1 T1D	8	F	MIAA, GAD65, ICA, ZNT8	Normal	1
9	Stage 1 T1D	41	F	GAD65, ICA	Normal	1
10	HC	13	F	None	ND	Non-diabetic
11	HC	17	F	None	ND	Non-diabetic
12	HC	9	M	None	ND	Non-diabetic
13	HC	17	F	None	ND	Non-diabetic
14	HC	17	F	ND	ND	Non-diabetic
15	HC	14	M	ND	ND	Non-diabetic
16	HC	46	M	ND	ND	Non-diabetic
17	HC	48	F	ND	ND	Non-diabetic

a Healthy controls (HC) are first-degree relatives of a T1D individual.

b At initial screening visit, TrialNet Pathway to Prevention study participants are screened for MIAA/GAD65/IA2 autoantibodies only. If autoantibody positive, participants are screened for ICA and ZNT8.

c Islet autoantibody and oral glucose tolerance test (OGTT) results were obtained at the time of blood draw and PBMC collection.

d Participant 2 initially screened positive for ≥2 islet autoantibodies but was only single positive for GAD65 at PBMC collection.

e Normal OGTT results were defined as follows for blood glucose measurements: fasting < 110 mg/dl, 30, 60, or 90 min < 200 mg/dl, and 2 h < 140 mg/dl.

f ND: Not determined. Four HC individuals were obtained outside of Type 1 Diabetes TrialNet and were not screened for islet autoantibody positivity at time of blood draw. HCs were not screened by OGTT.

### Participant selection and clinical information

All participants were recruited at Vanderbilt University Medical Center, with most participants recruited via the Type 1 Diabetes TrialNet Pathway to Prevention study. Participants included people who met inclusion criteria listed in Table S1. We defined Stage 1 T1D individuals as those who (1) initially screened positive for 2 or more islet autoantibodies (against MIAA, GAD65, IA2, ICA, and ZNT8) via TrialNet testing and (2) had normal OGTT results (blood glucose, mg/dl: fasting < 110; 30, 60, or 90 min < 200; 2 h < 140) on the date of blood draw. Age/sex-matched individuals who tested negative for MIAA, GAD65, and IA2 at initial TrialNet screening or non-diabetic first-degree relatives of T1D individuals identified outside of TrialNet were used as healthy controls. Participant demographics are shown in Table 1. Non-identifying participant metadata were managed using the REDCap software system (Vanderbilt).^33^

### Sample collection and processing

Peripheral blood mononuclear cells (PBMCs) were obtained by collecting whole blood into Vacutainer CPT Mononuclear Cell Preparation Tubes with sodium citrate (BD) via peripheral venipuncture. Cells were washed in phosphate buffered saline (PBS) and red blood cells were lysed using ACK Lysis Buffer (Gibco). Cells were washed twice more in PBS and counted. Cells were resuspended and cryopreserved in 10% dimethyl sulfoxide (DMSO) in heat-inactivated fetal bovine serum (FBS) at a rate of -1°C/minute until they reached a temperature of −80°C for 24–72 h, after which they were transferred to liquid nitrogen storage.

### CITE-seq labeling and flow cytometry sorting

PBMCs were rapidly thawed, washed in Hank’s balanced salt solution (HBSS), and resuspended in flow cytometry staining buffer (1× PBS, 0.02% EDTA, 1% heat-inactivated FBS). Cells were stained on ice with antibodies reactive against CD19-PE (SJ25C1, Tonbo Biosciences) and CD3-eflour450 (OKT3, Biolegend), plus an amine-reactive viability dye (NHS ester) conjugated to Alexa 700 (ThermoFisher Scientific), along with the oligo-tagged CITE-seq antibody panel outlined in Table S3. Each sample was stained with hash 1, 2, or 5 antibodies (BioLegend) for multiplexing. CD19^+^ CD3^−^ viable cells were flow cytometry purified using a BD FACSAria III in the VUMC Flow Cytometry Shared Resource.

### Single-cell profiling of B cells

Three samples (including at least 1 sample per disease group) stained with separate hash antibodies were combined and delivered to the VANTAGE Shared Resource for library preparation and sequencing. The Chromium Next-GEM Single Cell 5' Library & Gel Bead Kit, Chromium i7 Multiplex Kit, Chromium Single Cell A Chip kit, Chromium Single Cell V(D)J Enrichment (Human B Cell) Kit, and Chromium Single Cell 3'/5' Library Construction Kit (10× Genomics) were used for library preparation and sequence amplification via a 10X Chromium Controller instrument. 5,000 cells were targeted per sample (15,000 cells per lane). A target of 50,000 reads per cell was sequenced using an Illumina NovaSeq6000 (S4) PE150. Data were de-multiplexed and processed using CellRanger v4.0.0 (10× Genomics) using GRCh38 as reference.

### Analysis of differential gene expression (RNA-seq), phenotypic (CITE-seq) and immune repertoire (BCR-seq) single-cell data

Custom R scripts using Seurat package (v5.1.0) were used to simultaneously analyze RNA-seq, CITE-seq, and BCR-seq data. Data were demultiplexed based on hashtag antibodies using the HTODemux function. Cells with fewer than 200 RNA features and/or that contained >10% mitochondrial genes were removed. RNA-seq data were normalized and scaled using the SCTransform function. CITE-seq data were normalized using NormalizeData with centered-log ratio (CLR) transformation. Data from individual lanes were integrated using SelectIntegrationFeatures, PrepSCTIntegration, FindIntegrationAchors, and IntegrateData functions in Seurat. B cell subset identities were manually assigned to individual Seurat-determined clusters based on transcriptional profiles that were consistent with other studies defining these populations.^34–36^ Immunoglobulin VH, Vκ, Vλ genes were regressed out to prevent them from driving cluster assignments using FindNeighbors and FindClusters.

The Seurat FindMarkers function, which uses a non-parametric Wilcoxon rank-sum test, was used to identify differentially expressed genes (defined as fold change >1.2 in line with other single-cell RNA-seq studies in B cells,^37–39^ adjusted *P* value < 0.05, and expressed in > 30% of the cells in either group) between Stage 1 T1D and control groups within each transcriptionally defined cluster. VH, Vκ, Vλ, JH, Jκ, Jλ, Cκ, Cλ, and Y-chromosome genes were omitted from differential gene analysis. Venn diagrams showing shared differentially expressed genes were generated using the VennDetail package in R (v. 1.18.0). See Supplemental Files, Tables S5–S21 for differential gene analysis gene lists.

CellRanger v4.0.0 (10X Genomics) was used to filter on cells with high confidence VDJ calls and remove BCRs that were unproductive, not in-frame, or lacked CDR3s and assign V, D, and J gene segment, isotype, and clonotype identity (clonotypes defined as B cell clones that expressed matching heavy and light variable genes with ≥ 85% CDR3 identity). Another study looking at B cells in peripheral blood and cerebrospinal fluid in multiple sclerosis patients also used *n* ≥ 2 clones per clonotype as a definition of clonally expanded B cells.^40^ Thus, we defined clonally expanded B cells as *n* ≥ 2 clones (minimum 0.09% of total B cells per donor). VDJ data from CellRanger were run through IMGT HighV-QUEST^41^ which was used to determine % somatic hypermutation in VH and VL gene segments.

### Pathway analysis

Pathway enrichment was performed on upregulated genes in Stage 1 T1D memory B cells compared to healthy memory B cells using g: Profiler^42^ as previously described (https://baderlab.github.io/CBW_Pathways_2021/CANgprofiler-lab.html). Upregulated genes in Stage 1 T1D memory B cells with a fold change > 1.2 and a *P*_adj_ < 0.05 were included in the gene list query, and VH, VL, and HLA genes were stripped from pathway analysis to avoid skewing (*n* = 171 total genes). The analysis was run as a non-ordered query using the Benjamini–Hochberg false discovery rate (FDR) significance threshold set to 0.05 and included the data sources GO molecular function and GO biologic process without electronic GO annotations. Only gene sets containing 10–1,000 terms were included in analysis.

### Recombinant antibody expression and purification

A subset of clonally expanded Stage 1 T1D BCRs were recombinantly expressed as monoclonal antibodies (mAb) as follows. Heavy and light chain fragments of interest were commercially synthesized with flanking restriction enzyme sites (EcoRI and XhoI, IgG1; EcoRI-HF and BsiWI, IgK; EcoRI-HF and AcrII, IgL) (Twist Biosciences) and cloned into the corresponding sites in IgG1, IgK, or IgL expression vectors (Twist Biosciences). Expi293F cells (Gibco) were cultured in Freestyle F17 Media (Gibco) supplemented with GlutaMax (Gibco) and 0.1% Pluronic F-68 (Gibco). Paired heavy and light chain vectors (0.65 µg/ml) were combined in Opti-MEM media (Gibco) then incubated with polyethylenimine (PEI) transfection reagent (Kyfora Bio) at a final concentration of 3 µg/ml and transfected into Expi293F cells (Gibco) at ∼2 million cells/ml. Cells were cultured at 5% CO_2_ saturation and 37 °C with shaking. After 5 d, cells were harvested, spun at 2,300 rpm × 20 min, and filtered with Nalgene Rapid Flow filter with 0.45 um PES membrane. Expressed mAb were purified using Protein A resin (GenScript #L00210). The column was washed with 1× PBS, and mAb was eluted with 0.1M glycine-HCL pH 3.0, then neutralized with 1M Tris pH 8.0. Eluted mAb was dialyzed into 1× PBS and concentrated using a 10 kDa Amicon Ultra centrifugal filter. Alternatively, BCRs were commercially recombinantly expressed as mAb, purified and buffer exchanged by Genscript using the Genscript TurboCHO-HT 2.0 expression system. Purified mAb concentration was determined by A280.

As an anti-insulin human mAb positive control, we designed a recombinant mAb expressing the heavy chain and light chain variable regions from the validated anti-insulin murine mAb125^43^^,^^44^ in a human IgG1 and IgK expression vector, respectively. This mAb was expressed and purified by Genscript, as above.

### Enzyme-linked immunosorbent assay (ELISA)

For anti-insulin ELISAs, Maxisorp 384-well ELISA plates were coated with human insulin (Sigma #I2643) at 1 μg/ml in borate buffered saline, pH 8 overnight at 37 °C. Plates were blocked with 0.5% chicken serum in 1× PBS (0.5% CS 1× PBS) for 1 h at room temperature and washed 5X with 1/2X PBS. Plates were incubated at room temperature with recombinant experimental and control mAbs, serially diluted 1:2 from 10 µg/ml in 0.5% CS 1× PBS plus 0.1% Tween (0.5% CS 1X PBS-T). Bound mAb was detected by goat anti-human IgG-HRP (SouthernBiotech 2040-05) diluted 1:2,500 in 0.5% CS 1× PBS-T. Plates were washed 5× with 1/2× PBS and TMB Ultra ELISA substrate (Thermo Fisher #34029) was added. Plates were read at O.D. 370 nm every 5 min from 10 to 30 min, using a microplate reader (BioTek Synergy LX multi-mode reader). For inhibitable insulin binding determination, parallel wells were incubated with or without 100 µg/mL insulin competitor prior to transfer to ELISA plate, using the protocol outlined above.

To test binding to insulin in solution, mAbs of interest were serially diluted 1:2 from 10 µg/ml in PBS and coated on the Maxisorp 384-well ELISA plate, and blocked as above. Biotinylated insulin, at 1 µg/ml, was added to the plate and detected by streptavidin-HRP (SouthernBiotech 7105-05) diluted 1:2,500 in 0.5% CS 1× PBS-T. Plates were washed, substrate was added, and plates were visualized as above. For ELISAs to promote avidity, plates were coated with 1 µg/ml streptavidin, washed, and 1 µg/ml biotinylated insulin was added. Plates were blocked and primary mAbs of interest, secondary mAbs and substrate were added as above.

### Human HEp-2 cell immunofluorescence

HEp-2 cell coated slides (BION ENTERPRISES LTD ANA (HEp-2) Test System, ANK-120) were incubated with purified mAb at 1 µg/ml or control sera in a moist chamber at room temperature for 30 min. Controls provided with the kit included anti-nuclear antibody (ANA)^+^ and ANA^−^ human sera. Slides were washed twice with PBS for 5 min and stained with FITC-goat anti-human Ig per the manufacturer’s instructions and incubated in a moist chamber at room temperature for 30 min. Slides were washed twice with PBS for 5 min, mounted with DAPI mounting medium (Southern Biotech 0100-20), and visualized by fluorescence microscopy (Olympus BX60 epifluorescence microscope coupled with a CCD camera and MagnaFire software (Optronics International) at 40× magnification. Image brightness and contrast were optimized using Adobe Photoshop. Images were blind scored for presence/absence of HEp-2 cell reactivity, using positive and negative human serum control staining to establish positivity thresholds.

### Statistical analysis

Standard statistical tests utilized for each experiment are indicated in the corresponding figure legends and significance values were calculated using stats package in base R v4.3.2 or GraphPad Prism v9.3.1 (GraphPad Software).

### Study approval

These studies were approved by the University of Miami and Vanderbilt Institutional Review Boards. Written informed consent was received prior to participation. Ancillary Study Approval was granted by Type 1 Diabetes TrialNet.

## Results

### B cell frequency differs across clusters in Stage 1 T1D individuals compared to healthy controls

**Figure 1.**
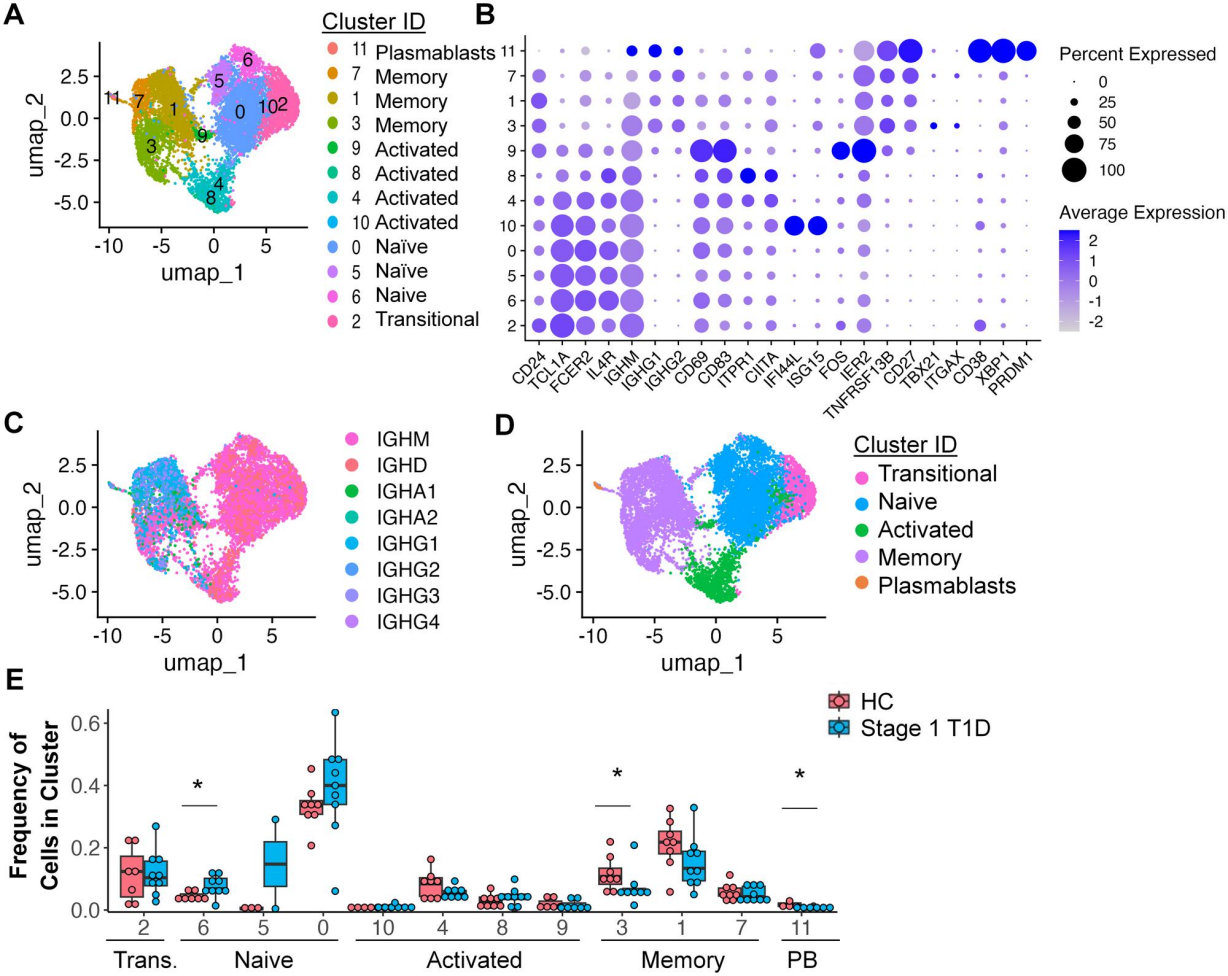
Stage 1 T1D individuals exhibit altered B cell cluster distribution compared to healthy controls. Single-cell RNA-seq was used to profile 2,000–5,000 purified CD19^+^ CD3^−^ cells isolated per donor from *n* = 9 stage 1 T1D and *n* = 8 healthy participants as in Methods. Seurat was used to identify 12 clusters which were further grouped as transitional, naïve, activated, memory, and plasmablast subsets based on manual inspection of isotype and gene expression profiles for each cluster. (A) RNA-seq cluster UMAP. (B) Dot plot of manually selected gene expression profiles used to assign B cell subsets to each cluster in panel A. (C) BCR isotype UMAP. (D) Collapsed subset UMAP. (E) Boxplots show the mean frequency of each B cell cluster by disease group; individual donors are plotted as points. A Wilcoxon rank sum test was used for all pairwise group comparisons of the frequency of cells per subset (healthy vs Stage 1), * *P* < 0.05, otherwise group comparisons were not significant (NS).

To investigate transcriptional, phenotypic, and immune repertoire changes within B cell subsets, we flow cytometry purified live, singlet, CD19^+^ cells from the peripheral blood of *n* = 9 Stage 1 T1D (≥2/5 islet autoantibodies, normal OGTT results) and *n* = 8 non-diabetic, first-degree relative (healthy) controls (Table 1), as in Methods. Seurat analysis of the *n* = 9,594 cells that met quality control criteria identified *n* = 12 unique clusters (Fig. 1A), which express distinct transcriptional profiles (Fig. S1). These twelve clusters were manually collapsed into five major B cell subsets (naïve, transitional, activated, memory, and plasmablast) defined by selected gene expression consistent with previous studies (Fig. 1B, 1D, Fig. S2).^35^^,^^38^ Memory B cells and plasmablasts exhibit class switching, as expected (Fig. 1C). To confirm that cluster identification was not skewed by donor or lane, frequency of cells from each donor and lane were analyzed to confirm that batch effects did not confound B cell clustering (Fig. S4).

The frequency of cells in each cluster was determined for each Stage 1 T1D and healthy control participant. The frequency of cells in naïve cluster 6 was significantly increased in Stage 1 T1D compared to healthy controls (*P* = 0.0417), while memory cluster 3 and plasmablast cluster 11 were significantly decreased in Stage 1 T1D compared to healthy controls (*P* = 0.0499 and *P* = 0.0190, respectively) (Fig. 1E). When the clusters were collapsed into their respective subset, the naïve subset maintained a marginally significant increase in frequency in the Stage 1 T1D individuals (*P* = 0.0464), while the plasmablast subset maintained a significant decrease in cell frequency in Stage 1 individuals compared to healthy controls (*P* = 0.0190) (Fig. S2). The total memory cluster was not significantly different in Stage 1 T1D vs. healthy controls (Fig. S2).

### Genes upregulated in Stage 1 T1D individuals relative to healthy controls are observed across several B cell subsets

**Figure 2.**
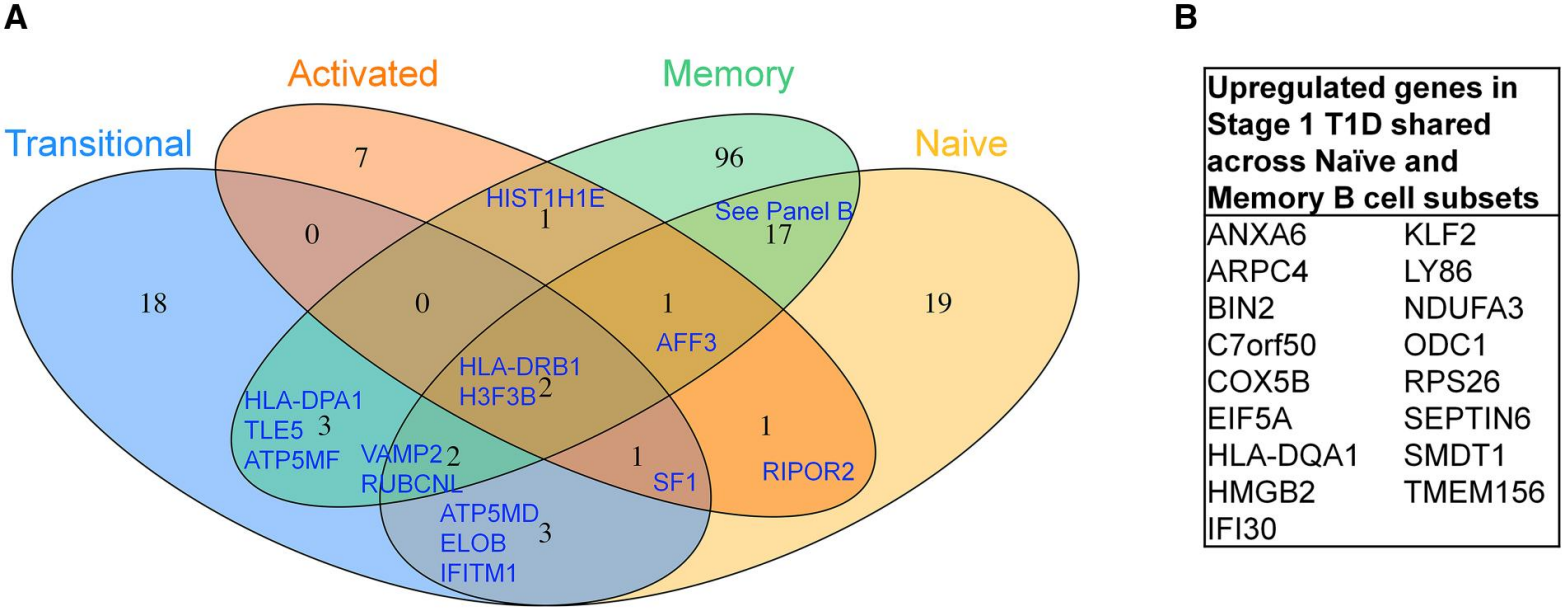
Shared gene expression changes are observed across B cell subsets in Stage 1 T1D individuals relative to healthy controls. Transitional, naïve, activated, and memory B cell subsets were identified as in Fig. 1D. (A) The Venn diagram indicates the number of upregulated genes in Stage 1 T1D (fold change >1.2, adjusted *P* value < 0.05, and expressed in ≥ 30% of cells in either disease group) within or across B cells subsets, relative to unaffected controls. Shared upregulated genes (blue text) are listed, with shared genes across the naïve and memory compartments (*n* = 17) listed in Panel B.

**Figure 3.**
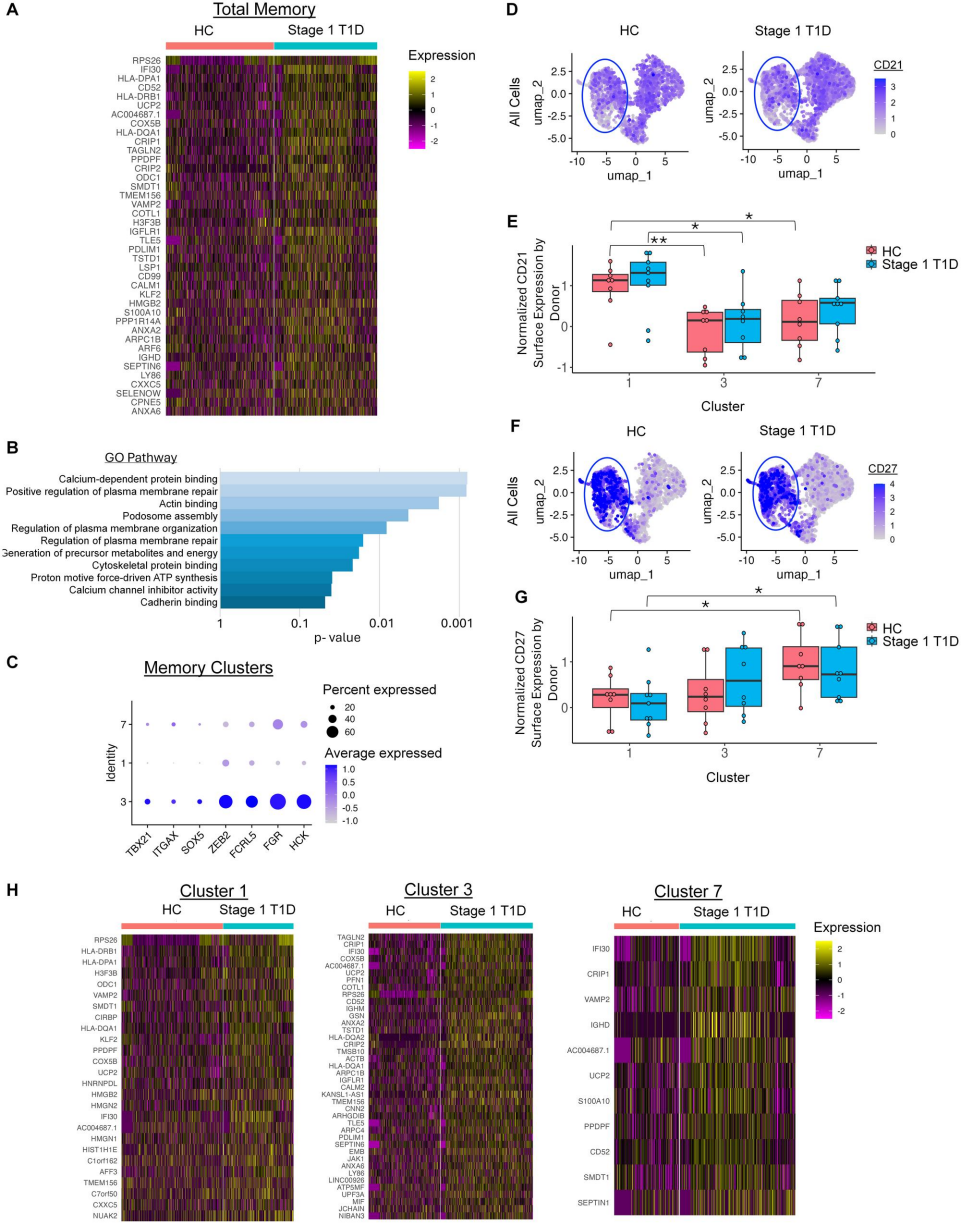
Total memory and atypical-like memory cluster 3 B cell compartments upregulate genes related to actin cytoskeleton rearrangement and antigen presentation in Stage 1 T1D individuals. Memory B cells were identified as in Fig. 1. (A) The top 40 upregulated genes in memory B cells in Stage 1 T1D (teal) compared to healthy control (peach) individuals are shown (fold change >1.2, adjusted p value < 0.05, and expressed in > 30% of cells in either disease group). (B) gProfiler pathway analysis performed on selected upregulated genes (fold change >1.2, adjusted p value < 0.05) identified enriched GO Ontology gene pathways in Stage 1 T1D individuals. (C) Genes that define atypical B cells were manually selected and shown for each memory B cell cluster (plot derived from all individuals concatenated), with size correlating with percent of cells expressing the gene and color correlating with level of expression. (D–G) CITE-seq analysis identified (D) CD21 surface expression on healthy and Stage 1 T1D B cells, quantified in panel (E), and (F) CD27 surface expression, quantified in panel (G). (H) The top 40 upregulated genes in Stage 1 T1D memory clusters 1, 3 and 7 compared to healthy controls are displayed as in panel A. A Wilcoxon rank sum test was used for all pairwise group comparisons of the normalized expression per cluster (HC vs Stage 1), * p < 0.05, ** *P* < 0.01, otherwise groups comparisons were NS.

Single nucleotide polymorphisms (SNPs) involved in B cell signaling and differentiation (*PTPN22, BACH2, PTPN2,* and *SH2B3*) were previously associated with T1D risk.^45^ We therefore sought to determine whether gene expression changes related to B cell signaling may be present within, and potentially across, specific B cell subsets in Stage 1 T1D vs. healthy individuals. The memory subset exhibited the highest number of upregulated genes (fold change >1.2, adjusted *P* value < 0.05, and expressed in > 30% of cells in either disease group) in Stage 1 T1D vs. healthy individuals (122 genes), with upregulated genes also observed in naïve (46 genes), transitional (29 genes), and activated (13 genes) B cell subsets. No significantly upregulated genes were identified in plasmablasts due to the small number of cells recovered (Fig. 2 and Fig. S5). We next identified upregulated genes in Stage 1 T1D individuals that were shared among at least two B cell subsets. Multiple major histocompatibility complex (MHC) class II genes involved in antigen presentation (*HLA-DPA1, HLA-DRB1, and HLA-DQA1*) were upregulated across Stage 1 T1D B cell subsets (Fig. 2), as was observed in autoantibody positive and Stage 3 T1D individuals.^17^^,^^46^ Metabolic genes involved in oxidative phosphorylation (*COX5B, ATP5MF, ATP5MD*, and *NDUFA3*) were upregulated in Stage 1 T1D transitional, naïve, and memory B cells compared to healthy controls. Additional genes involved in BCR signaling, including *RACK1, CDC42*, and *PTPRC* were uniquely upregulated in Stage 1 T1D transitional, naïve, and activated B cells, respectively (Fig. 3A, Figs. S5, S6).

### Memory B cells from Stage 1 T1D individuals upregulate genes implicated in BCR signaling, actin rearrangement and mitochondrial metabolism compared to memory B cells from healthy controls

**Figure 4.**
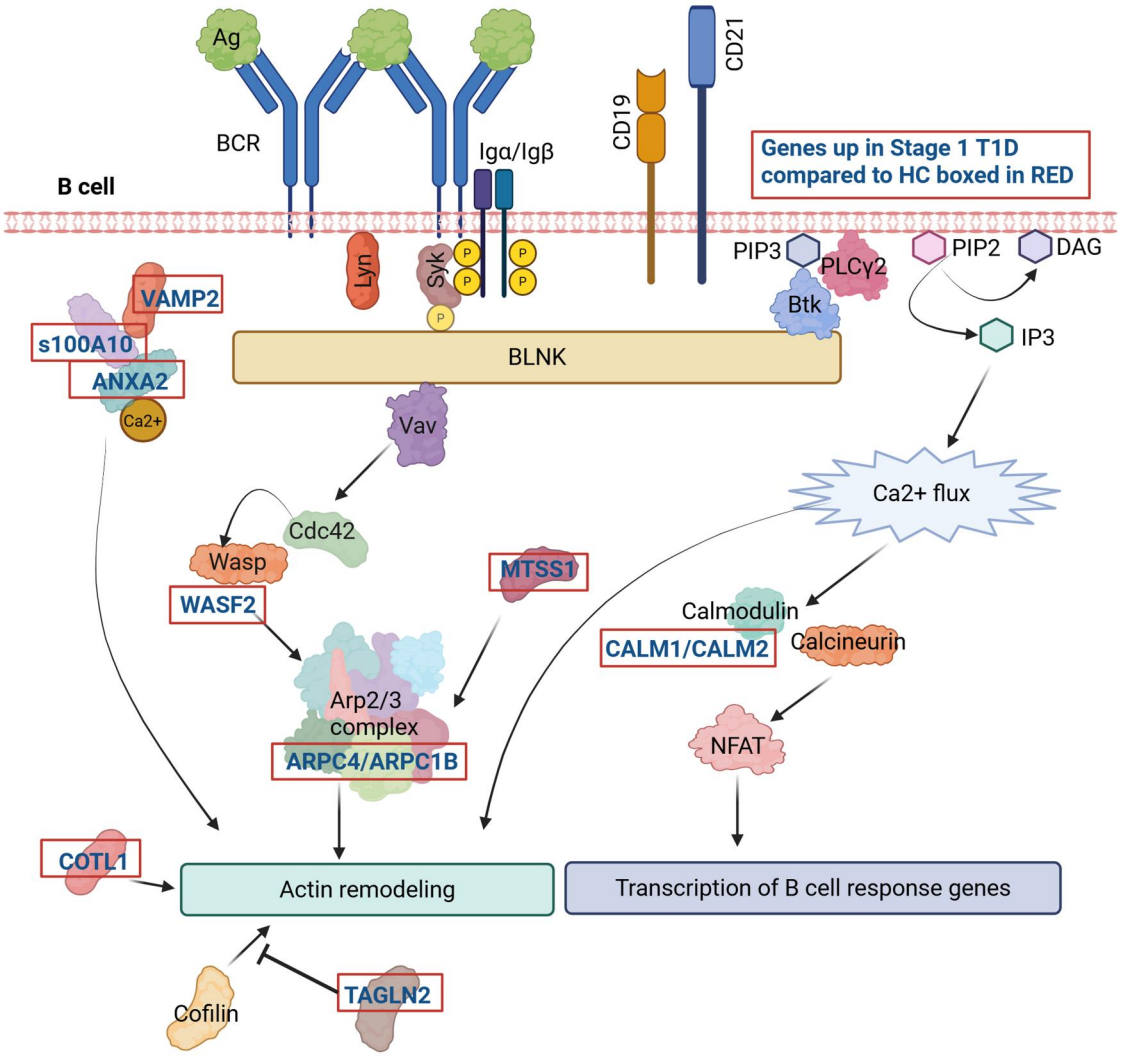
Stage 1 T1D memory B cells upregulate genes involved in BCR-mediated actin remodeling and calcium signaling. Modified from Bhanja et al.^60^ Red boxes indicated genes identified in this study as being upregulated in Stage 1 T1D relative to healthy control memory B cells as in Fig. 3 and Table S8. Figure made in BioRender.

Given that the Stage 1 T1D memory B cell compartment appeared most transcriptionally distinct compared to healthy controls, we identified enriched gene pathways and specific genes upregulated in this compartment (Fig. 3B, Table S2). Calcium-dependent protein binding (GO:0048306), actin binding (GO:0003779), and plasma membrane organization and repair (GO:1905686, GO:1903729, GO:1905684) pathways were enriched in the Stage 1 T1D memory B cells (Fig. 3B). Genes involved in these pathways are implicated in BCR-mediated actin rearrangement and signaling, as depicted in Fig. 4. The generation of precursor metabolites and energy pathway (GO:0006091) and proton motive force-driven ATP synthesis pathway (GO:0015986) and related genes (*UCP2, COX5B, SMDT1*, *MCUB,* ATP synthase genes: *ATP5F1C, ATP5MF, ATP5MC3*, and NADH dehydrogenase/complex I genes: *NDUFA7, NDUFS5, and NDUFA3*) were also enriched in Stage 1 T1D memory B cells compared to healthy controls (Fig. 3B).

**Figure 5.**
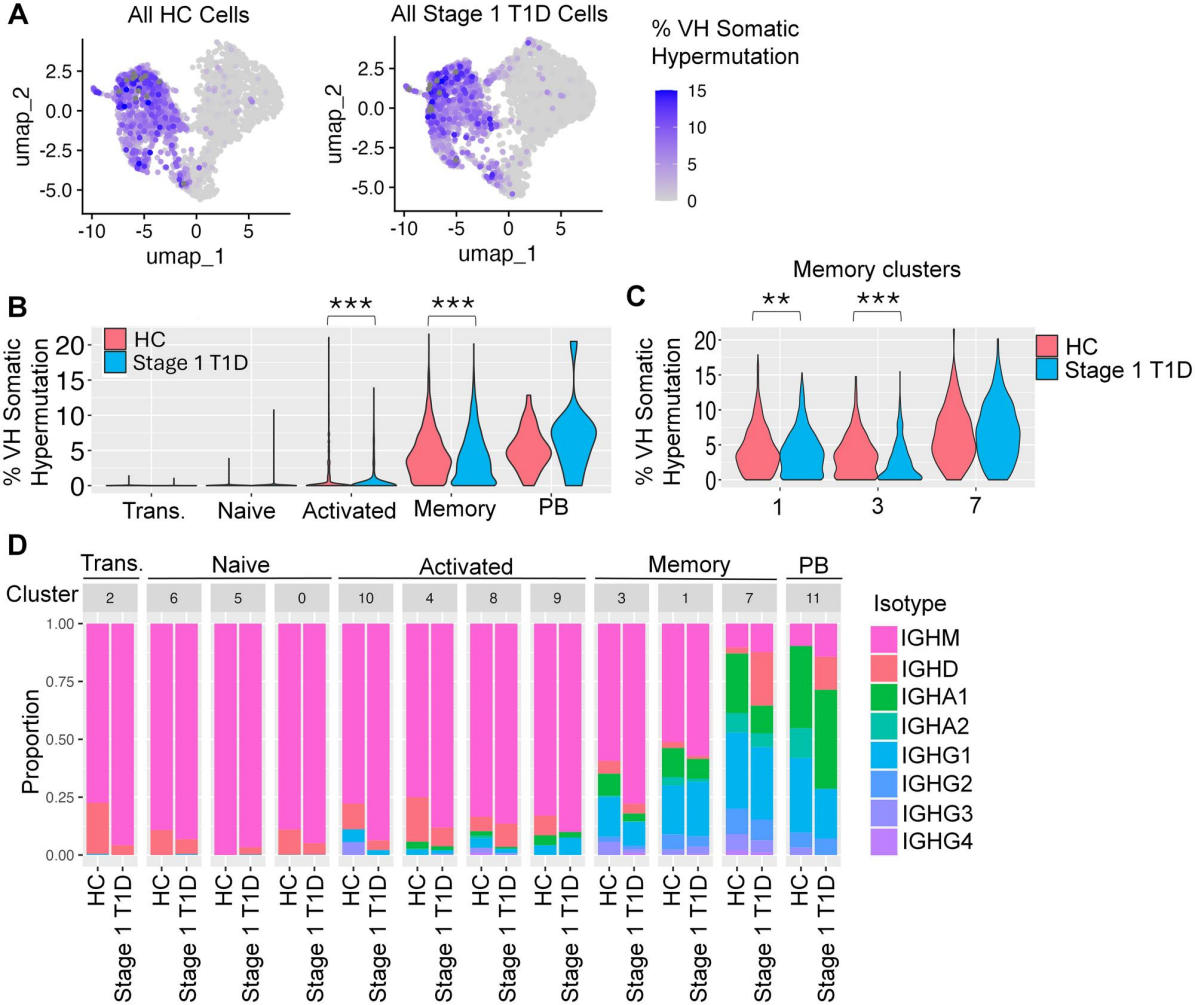
Total memory B cells from Stage 1 T1D individuals exhibit significantly decreased VH somatic hypermutation and atypical-like memory B cells experience limited class switching. Single-cell BCR-seq technology was used to profile the cells in Fig. 1. An integrated Seurat/IMGT/HighV-QUEST analysis pipeline was developed to generate (A) a UMAP of percent VH gene somatic hypermutation, quantified as violin plots in panel (B) and (C). (D) Isotype proportion per cluster is plotted for both disease groups. A Wilcoxon rank sum test was used for all pairwise group comparisons of percent mutation per subset or cluster (HC vs Stage 1 T1D), ** *P* < 0.01, *** *P* < 0.001, otherwise group comparisons were NS.

In addition to transcriptional analysis, we used CITE-seq to investigate surface expression of key B cell phenotypic markers and confirm B cell subset assignments for the transcriptionally defined clusters (Table S3, Fig. S3). Low CD21 expression on B cells has been associated with an atypical B cell phenotype and has been observed in other autoimmune diseases including rheumatoid arthritis, systemic lupus erythematosus, systemic sclerosis, and antisynthetase syndrome.^47–50^ Memory cluster 3 upregulated genes that define atypical memory B cells, including *TBX21* (Tbet), *ITGAX* (CD11c), *SOX5, ZEB2, FCRL5, FGR,* and *HCK*^51^ (Fig. 3C) and exhibited low CD21 surface expression compared to memory cluster 1 (Fig. 3D, 3E). Unlike double negative 2 (DN2) B cells, memory cluster 3 cells maintained surface CD27 expression (Fig. 3F, 3G) and were mostly non-class switched (Fig. 5D). We termed cluster 3 the “atypical-like” memory cluster.

Given the transcriptional and phenotypic heterogeneity across the 3 memory clusters identified (Fig. S7), we analyzed differentially expressed genes within each memory cluster across Stage 1 T1D and healthy controls (Fig. 3H). To emphasize the most consistent gene expression changes, genes were removed if they were not expressed in at least 30% of the cells in either disease group. We observed *n* = 26, 72, 11 upregulated genes in T1D Clusters 1, 3, and 7, respectively. Stage 1 T1D memory B cells in clusters 1, 3, and 7 upregulated many of the same genes represented in the total memory subset, with some genes being uniquely upregulated in each cluster. Stage 1 T1D B cells in cluster 1 uniquely upregulated genes *HMGB2, HMGN2,* and *HMGN1* involved in chromatin structure and transcription, compared to healthy individuals (Fig. 3H). Stage 1 T1D memory B cells in cluster 3 uniquely upregulated *JCHAIN*, which may suggest these cells are poised to become antibody secreting cells, while Stage 1 T1D memory B cells in cluster 7 upregulated *SEPTIN1,* implicated in lymphocyte motility (Fig. 3H).

### The memory B cell compartment from Stage 1 T1D individuals exhibits minimal BCR somatic hypermutation and atypical-like memory B cells undergo limited class switching

We next investigated BCR repertoire differences between Stage 1 T1D and healthy individuals using an integrated Seurat/IMGT/HighV-QUEST pipeline that we designed and applied to scBCR-seq data. Variable heavy chain (VH) somatic hypermutation and isotype switching were low in transitional, naïve, and activated B cell subsets, with higher levels observed in memory B cells and plasmablasts, as expected (Fig, 5A, 5B, 5D). Mean VH somatic hypermutation in the memory subset was significantly decreased in Stage 1 T1D individuals (3.89%) compared to healthy controls (4.29%) (Fig. 5B). Specifically, atypical-like memory cluster 3 exhibited decreased mean VH somatic hypermutation in Stage 1 T1D individuals (2.42%) compared to healthy controls (3.52%). Memory cluster 1 also exhibited decreased VH mutation in Stage 1 T1D B cells (3.89%) compared to healthy controls (4.31%), although to a less significant extent (Fig. 5C). Decreased VH somatic hypermutation was also observed in the Stage 1 T1D activated subset (0.622%) compared to healthy controls (1.11%) (Fig. 5B). Atypical-like memory cluster 3 exhibited a higher proportion of IgM B cells in Stage 1 T1D individuals compared to healthy controls (Fig. 5D).

### Clonally expanded memory B cells contain insulin binding specificities

**Figure 6.**
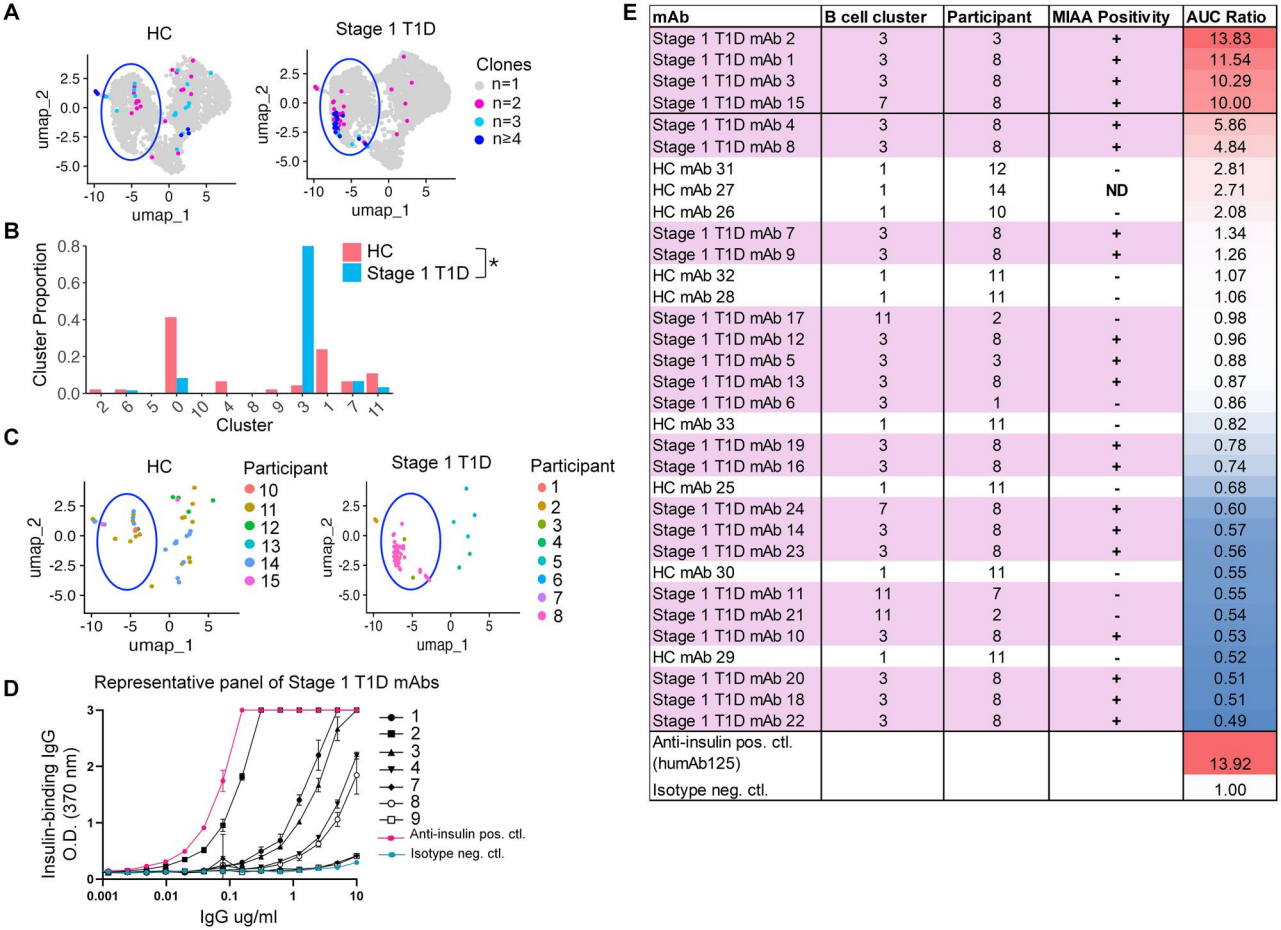
Clonally expanded memory B cells are a reservoir for insulin-binding B cells in Stage 1 T1D individuals. As in Fig. 5, BCR-seq data and the Seurat/IMGT/HighV-QUEST pipeline was used to identify clonally expanded cells, defined as *n *≥ 2 B cells with matching heavy and light chain V genes and at least 85% CDR3 identity. (A) UMAP of clonally expanded cells, color represents the extent of clonal expansion in the dataset. Blue circle notes the memory compartment. (B) Distribution of clonally expanded cells across clusters was assessed by Pearson’s χ^2^ test (HC vs Stage 1 T1D), *P* < 0.0477. (C) UMAP of participant assignments for each clonally expanded cell. (D) Clonally expanded BCRs, recombinantly expressed as IgG1 mAb, were serially diluted from 10 µg/ml and screened for insulin binding by ELISA. A representative panel of *n* = 7 BCRs from clonally expanded Stage 1 T1D cells is shown. Anti-insulin positive control (pink) is a validated anti-insulin mouse BCR (mAb125) expressed as human IgG1. Negative control (blue) is a viral-specific human IgG1 isotype negative control. Outliers identified by Prism ROUT analysis (Q = 1%) among *n* = 4 technical replicates were removed. Mean and standard deviation are represented. (E) *n* = 24 Stage 1 T1D BCRs and n = 9 HC BCRs were screened as in panel D. AUC was determined for insulin binding ELISA curves and compared to the AUC for the isotype negative control. AUC ratio values are displayed as a heat map, with Stage 1 BCRs highlighted in pink. Positive cutoff for insulin binding was set at half the AUC ratio of the anti-insulin positive control, an AUC ratio of 7.

Clonally expanded B cells, defined as *n* ≥ 2 B cells with matching heavy and light chain V genes and at least 85% CDR3 identity, were identified in both Stage 1 T1D and healthy individuals, with extent of clonal expansion indicated (Fig. 6A). Clonally expanded B cells were differentially distributed across B cell subsets in Stage 1 T1D individuals compared to healthy controls (Fig. 6B). In the Stage 1 T1D group, clonally expanded B cells were predominantly observed in the memory subset, and particularly in the atypical-like memory cluster 3 (Fig. 6B). Further investigation revealed that Stage 1 T1D participant 8 heavily contributed to the clonal expansion observed in the memory subset, with lesser contribution from Stage 1 T1D participants 1 and 3 (Fig. 6C). Stage 1 T1D participant 8 was an 8-year-old female, who screened positive for microinsulin autoantibody (MIAA), glutamic acid decarboxylase 65 (GAD65) autoantibody, islet cell autoantigens (ICA) autoantibody, and zinc transporter 8 (ZNT8) autoantibody at the time of blood draw (Table 1). Interestingly, the clonally expanded memory B cells identified in healthy individuals were largely found in memory cluster 1, not atypical-like memory cluster 3 (Fig. 6B).

We hypothesized that clonally expanded B cells may serve as a reservoir for anti-insulin B cells in early stage T1D individuals. To test this, we recombinantly expressed a panel of clonally expanded BCRs from Stage 1 T1D individuals and healthy controls from the memory (and plasmablast) subsets as monoclonal antibody (mAb) and screened for binding to insulin by ELISA (Fig. 6D, Fig. S8). For a given clonotype, we prioritized BCR expression of clonally expanded B cells found in the memory or plasmablast subset, for which percent VH somatic hypermutation was the highest. These B cells ranged in frequency from *n* = 2 to *n* = 12 clones per clonotype (Table S4). Anti-insulin mouse BCR (mAb 125) expressed as human IgG1 (humAb 125) was designed as a positive control, as the murine version of this mAb supports diabetes development in NOD mice.^52^ We used a human anti-viral IgG1 negative control.^53^ The insulin-binding positive control mAb showed an ∼ 14-fold increase in insulin ELISA area under the curve (AUC) compared to the isotype negative control (Fig. 6E). Therefore, we set the positive cutoff for insulin binding at half the anti-insulin positive control mAb AUC ratio, or 7-fold greater than the isotype negative control. Four Stage 1 T1D mAbs met this cutoff, with Stage 1 T1D mAb 2 exhibiting the highest AUC ratio of 13.83, comparable to the anti-insulin positive control mAb, with an AUC ratio of 13.92 (Fig. 6E). No healthy control mAbs met this cutoff, with the highest AUC value of 2.81 (HC mAb 31) (Fig. 6E). The four anti-insulin mAbs were isolated from two Stage 1 T1D participants (22% of the Stage 1 T1D participant group). This aligns with previous murine studies, which identified detectable populations of anti-insulin B cells in the pancreas of only ∼30% of NOD mice.^54^

### Anti-insulin BCRs isolated from Stage 1 T1D individuals are minimally mutated and display avidity-dependent binding to insulin

The majority of published human anti-insulin BCR sequences were isolated from people with established T1D (Stage 4 T1D) who were on insulin therapy, which is known to elicit a “foreign” anti-insulin antibody response to exogenous insulin administration, even in people who were previously insulin autoantibody negative.^26–30^ The four insulin-binding mAbs isolated here, from insulin-therapy naïve Stage 1 T1D individuals, used various heavy and light chain genes, with IGHV3-15 expressed in 3/4 mAbs and no overlap in variable light chain (VL) genes (Table 2). Percent somatic hypermutation of the VH gene ranged from 0.35% to 1.68% (Table 2), in line with the limited BCR mutation observed in published anti-insulin BCRs.^17^^,^^26–29^ Stage 1 T1D mAb 2, which exhibited the highest insulin binding AUC ratio, exhibited the lowest VH gene somatic hypermutation (0.35%), but these mutations resulted in two amino acid replacements in the V gene complementarity determining regions (CDRs). Only Stage 1 T1D mAb 1 exhibited no amino acid replacements across the V gene CDR regions of the heavy and light chain. All four insulin-binding mAbs were isolated from non-class-switched, memory B cells, with three in cluster 3 and one in cluster 7 (Table 2). Consistent with these findings, we previously showed that insulin-binding B cells skew toward the memory subset in presymptomatic T1D individuals using high throughput screening of PBMC cultures stimulated to support antibody-secreting cell differentiation, coupled with ELISA detection of wells containing insulin antibody-secreting clones.^55^

**Table 2.** BCR gene usage and somatic hypermutation of anti-insulin mAbs isolated from Stage 1 T1D individuals.

mAb^a^	Insulin-binding AUC ratio	Disease	VH gene	VH gene % SHM	# VH CDR AA replacements	VL gene	VL gene % SHM	# VL CDR AA replacements	Isotype	Cluster
1	11.54	Stage 1 T1D	IGHV3-15	0.68	0	IGKV3-11	0.36	0	IGHM	3
2	13.83	Stage 1 T1D	IGHV3-33	0.35	2	IGLV2-14	0.35	2	IGHM	3
3	10.29	Stage 1 T1D	IGHV3-15	1.02	0	IGKV1-5	0.72	1	IGHM	3
15	10.00	Stage 1 T1D	IGHV6-1	1.68	2	IGKV1D-39	1.08	0	IGHD	7

a mAbs were included if they met the insulin-binding cutoff established in Fig. 6E.

**Figure 7.**
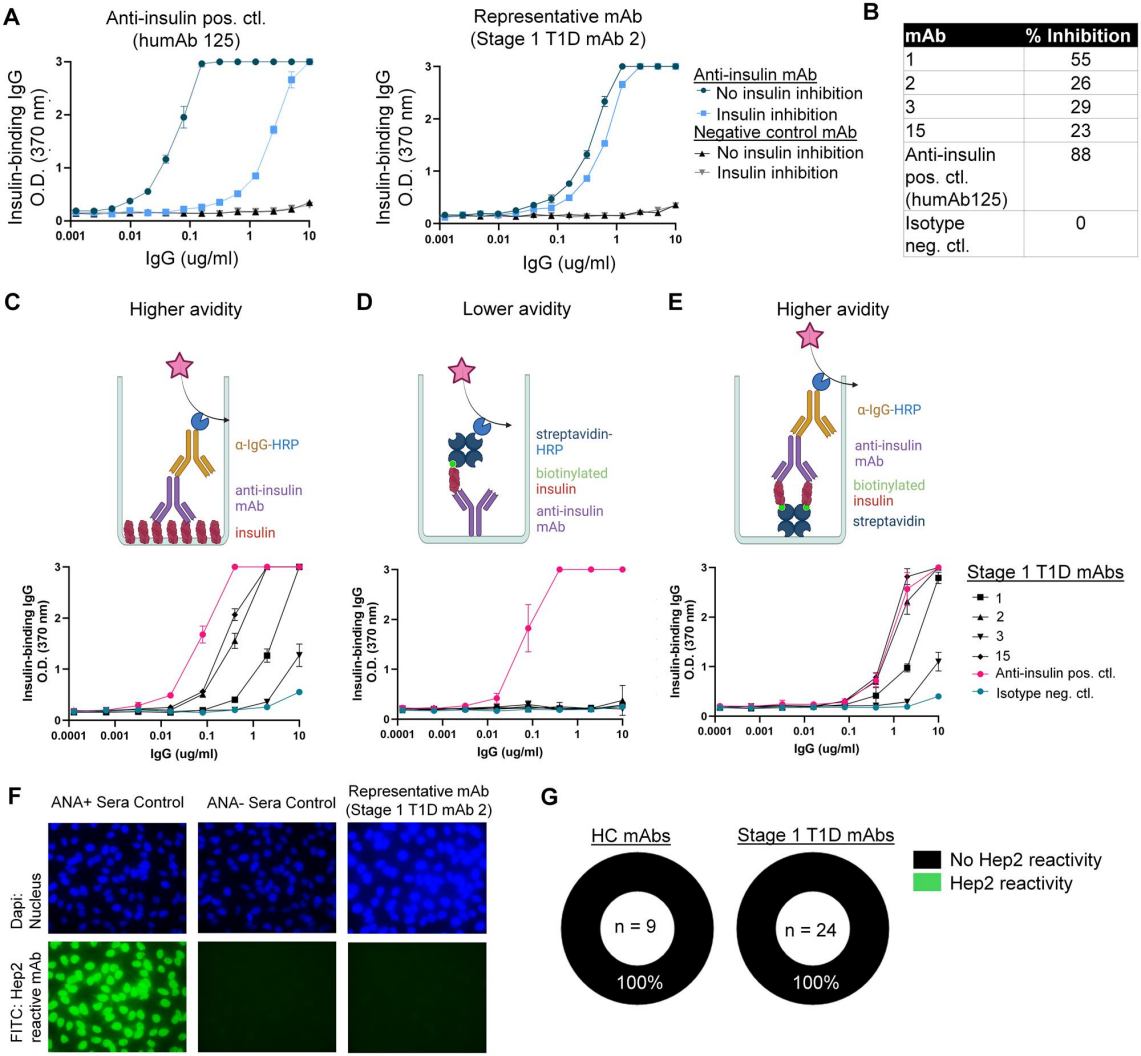
Insulin-binding BCRs isolated from Stage 1 T1D individuals show avidity-based binding to insulin and do not exhibit polyreactivity to human HEp-2 autoantigens. (A) mAbs that met positivity for insulin binding cutoffs in Fig. 6 were screened for inhibitable insulin binding using 100X soluble insulin competitor as in Methods, with the same positive and negative controls as in Fig. 6. Mean and standard deviation are represented. (B) Percent inhibition was calculated by evaluating the AUC ratio of the inhibited insulin binding/un-inhibited binding. To investigate the impact of avidity on insulin recognition, mAbs were screened for binding to (C) an insulin-coated plate, (D) insulin in solution, and (E) biotinylated insulin bound to a streptavidin-coated plate, by ELISA. Outliers identified by Prism ROUT analysis (Q = 1%) among *n* = 4 technical replicates were removed. Mean and standard deviation are represented. (F–G) mAb reactivity against human HEp-2 cells was screened by immunofluorescence as in Methods. Anti-Ig-FITC (green) secondary was used to detect mAbs bound to HEp-2 cell antigens, with DAPI (blue) counterstaining. (F) Representative images for the anti-nuclear autoantigen positive (ANA+) and negative (ANA-) serum controls and an insulin binding Stage 1 T1D BCR (mAb 2) are shown. (G) Summary of HEp-2 reactivity for each mAb tested in the Stage 1 T1D and HC groups. Cartoons in panels C-E were generated using BioRender.

Prior studies in NOD mice showed that the majority of insulin-binding B cells that arise spontaneously in the pre-diabetic repertoire are not high affinity; rather, they depend on avidity-based interactions with insulin autoantigen.^56^ Similarly, Viant et al. showed that memory B cells responding to immunization often rely on increased valency to recognize antigen.^57^ We therefore tested whether avidity-based interactions are responsible for the four anti-insulin BCRs isolated here, using several ELISA designs. First, inhibitable insulin binding was measured. The insulin-binding mAbs were incubated with a high concentration of soluble insulin, or buffer, before measuring plate-bound (higher avidity) insulin binding by ELISA (an example of which is shown in Fig. 7A). To quantify the amount of inhibitable binding observed, the AUC ratio of insulin binding with insulin inhibition compared to without insulin inhibition (buffer) was determined. Because some of the mAbs reached the maximum measurable insulin-binding optical density (OD) value, the AUC was only calculated from the concentration at which the highest OD or the first maximum measurable OD (OD = 3) was determined. As shown in Fig. 7B, inhibition of plate-bound insulin binding of these mAbs ranged from 23% to 55% inhibitable by soluble insulin competitor, compared to the anti-insulin positive control (originally derived from a mouse immunized with foreign insulin) that showed 88% inhibition of insulin binding. Second, to test whether avidity drives insulin recognition of these anti-insulin mAbs, rather than conformational changes of plate-bound insulin, we compared insulin recognition of the four anti-insulin mAbs to plate bound insulin (Fig. 7C), to insulin in solution (Fig. 7D), and to biotinylated insulin bound to a streptavidin-coated plate (Fig. 7E) by ELISA. All anti-insulin Stage 1 T1D mAbs and the anti-insulin positive control bound insulin on the plate (higher avidity) (Fig. 6D,  7C). Only the anti-insulin positive control showed insulin recognition to insulin in solution (lower avidity) (Fig. 7D). To promote avidity without directly binding insulin to the plate, long-chain biotinylated insulin was bound to an avidin-coated plate. All anti-insulin Stage 1 T1D antibodies and the anti-insulin positive control showed biotinylated insulin binding activity by ELISA (Fig. 7E). These data highlight a polyclonal anti-insulin B cell response in Stage 1 human T1D which is not highly mutated and which is avidity rather than affinity driven.

### Insulin-binding B cells isolated from Stage 1 T1D individuals do not show HEp-2 autoantigen polyreactivity

Autoreactive B cells can exhibit polyreactive binding to multiple antigens.^58^ Since the insulin-binding mAbs showed ≤ 55% inhibition of insulin binding with the addition of soluble insulin, we tested anti-insulin mAb polyreactivity to other antigens using a human HEp-2 cell immunofluorescence assay, with representative examples shown in Fig. 7F. None of the recombinant BCRs from Stage 1 T1D individuals or healthy controls exhibited HEp-2 autoreactivity (Fig. 7F, 7G, Fig. S9). Thus, the anti-insulin BCRs that we isolated from Stage 1 T1D individuals do not show broadly polyreactive binding profiles to Hep-2 cells, despite exhibiting avidity-based insulin binding.

### Insulin-binding B cells appear to upregulate genes involved in several key B cell functions compared to non-insulin-binding B cells in Stage 1 T1D individuals

**Figure 8.**
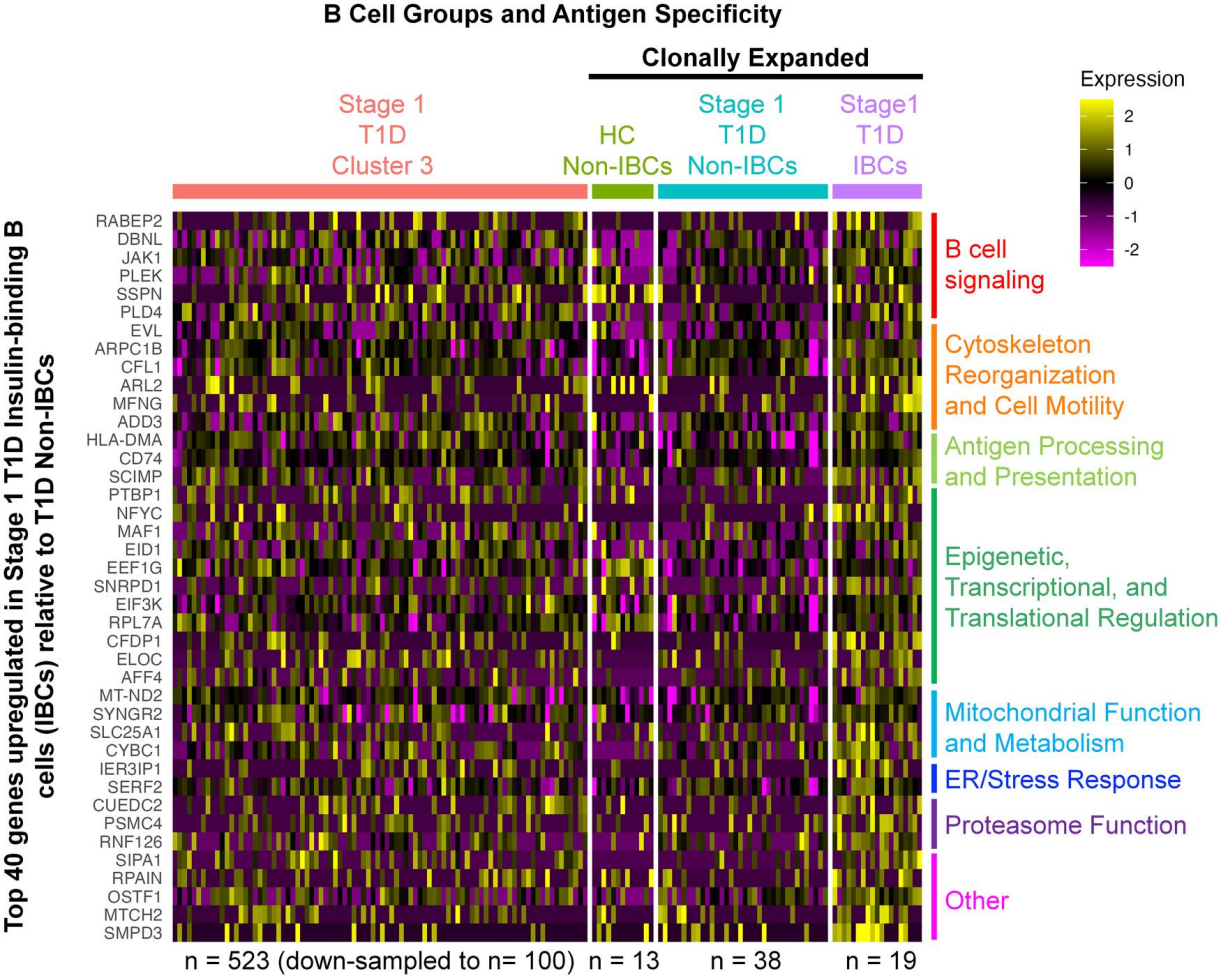
Insulin-binding B cells (IBCs) show a non-significant upregulation of genes related to B cell signaling and antigen presentation relative to non-IBCs in Stage 1 T1D individuals. Clonally expanded Stage 1 T1D or HC B cells that exhibited or did not exhibit insulin binding in Fig. 6, and all corresponding B cells which shared the same clonotype IDs and IgH and IgL CDR3 amino acid sequences were included in the Stage 1 T1D IBCs (purple, *n* = 19 cells), Stage 1 T1D Non-IBCs (teal, *n* = 38 cells) and HC Non-IBCs (green, *n* = 13 cells) groups respectively. Differential gene expression analysis was performed for Stage 1 T1D IBCs and Stage 1 T1D Non-IBCs. The top 40 upregulated genes were identified based on expression in at least 50% of Stage 1 T1D IBCs, and minimum 1.2-fold change. None of these genes showed adjusted *P* value < 0.05. All other B cells from Stage 1 T1D cluster 3 (peach, *n* = 523) were randomly down-sampled to *n* = 100 cells and included in the DEG heatmap.

To identify potential gene expression changes in insulin-binding B cells relative to non-insulin-binding B cells, we mined our RNA-seq data for our ELISA-validated insulin-binding and non-insulin-binding B cells (as defined in Fig. 6E) from Stage 1 T1D individuals. We included Stage 1 T1D B cells with identical clonotype ID and IgH and IgL CDR3 amino acid sequences to the four functionally validated insulin-binding B cells (*n* = 19 total cells) and 21 non-insulin-binding B cells (n = 38 total cells), as both groups were identified among clonally expanded B cells. Figure 8 shows a heat map of the top 40 genes with fold change ≥1.2 in Stage 1 T1D insulin-binding B cells (purple) compared to Stage 1 T1D non-insulin-binding B cells (teal), none of which reached statistical significance (defined as *P*  _adj_ value < 0.05). Gene expression in at least 50% of the insulin-binding B cell group was required to ensure a small fraction of the insulin-binding cells did not drive the identified DEGs. Healthy control non-insulin-binding B cells (green) are shown on the heatmap for comparison, as they were also derived from clonally expanded B cells. Lastly, given that most of the insulin-binding B cells had a cluster 3 identity, a down-sampled group of Stage 1 T1D memory cluster 3 cells (peach) was also included in the heatmap. Genes involved in B cell signaling, antigen presentation, and metabolism were identified as increased in insulin-binding B cells compared to non-insulin-binding B cells. From these data, we hypothesize that autoreactive B cells are transcriptionally distinct from non-autoreactive B cells in the same B cell cluster, which will need to be tested in the future.

Overall, our data highlight gene expression changes across several B cell subsets, and within specific clusters of B cells, identified in Stage 1 T1D individuals relative to healthy controls. These gene expression changes include upregulation of genes involved in key B cell biological processes including BCR-mediated actin rearrangement and signaling, metabolism, and antigen presentation. Here, we present methods to use transcriptionally based clustering combined with immune repertoire data to enrich for candidate autoantigen-specific BCRs. Characterization of these BCRs as recombinant mAb led to the identification of insulin-binding B cells in small volumes of blood (typically 2–5mL) isolated from Stage 1 T1D individuals. Application of single-cell technology for clinical monitoring is impractical currently, due to technical and cost barriers. However, implementation of these methods using larger human T1D cohorts could support more comprehensive characterization of autoreactive B cells in human T1D to lay a foundation to guide future biomarker development and immunotherapy design.

## Discussion

B cell-targeted therapy has shown temporary beta cell preservation in clinical trials for new-onset T1D individuals,^4^^,^^5^ as B cells are thought to present islet autoantigen to autoreactive T cells to promote T1D.^6–11^ Yet B cell changes that drive their pathogenic function at the earliest stage of human T1D, prior to beta cell dysfunction, are unclear. Here we report numerous peripheral blood B cell gene expression differences identified in Stage 1 T1D individuals compared to healthy controls within specific transcriptionally defined B cell clusters and subsets. To our knowledge, this is the first study to investigate combined gene expression and immune repertoire alterations focused on the earliest detectable stage of T1D (Stage 1) compared to healthy individuals. Stage 1 T1D individuals do not yet exhibit abnormal blood glucose regulation, based on OGTT results,^1^^,^^2^ helping to mitigate the effects dysregulated blood glucose may have on B cell gene expression. In addition, Stage 1 T1D individuals do not yet require exogenous insulin therapy, therefore avoiding the confounding impact that insulin therapy is known to have on B cell responses against insulin.^30^

Single-cell RNA-seq has been used previously to profile peripheral blood in T1D individuals, but analyses focused largely on non-B cell subsets, as reviewed in Hanna et al.^59^ The B cell subset-focused analysis presented here identified *n* = 122 genes in the memory subset, *n* = 26 of which were also upregulated in at least one other B cell subset. Several genes implicated in BCR signaling and actin rearrangement were upregulated in Stage 1 T1D memory B cells relative to healthy controls, including *ARPC1B, MTSS1,* and *TAGLN* (Figure 4). Actin rearrangement can positively and negatively regulate BCR signaling, as reviewed by Bhanja et al.^60^ Of note, deficiency in ARPC1B, a component of the Arp2/3 complex causes decreased F-actin, increased BCR diffusion, and increased tonic signaling in B cells.^61^ MTSS1 is an Arp2/3 activator, and deficiency in *MTSS1* led to defective BCR signaling in response to surface bound antigens.^62^ Transgelin 2 (*TAGLN2*) blocks actin depolymerization and was upregulated on activated B cells in lupus patients.^63^ Further studies are required to link these transcriptional changes in BCR signaling and actin rearrangement to functional differences. We cannot conclude whether the gene expression changes observed here are the cause or result of the underlying autoimmune process, or whether they are B cell intrinsic.

We observed decreased somatic hypermutation in the memory compartment, and particularly in the atypical-like memory cluster 3 of Stage 1 T1D individuals compared to healthy controls. Decreased somatic hypermutation was also reported amongst IgG^+^ peripheral blood B cells isolated from patients with early and established rheumatoid arthritis and Sjogren’s syndrome compared to healthy controls.^64^ Together, these findings suggest impaired BCR somatic hypermutation may be a generalized feature of human autoimmunity. It is currently unclear whether this reduced somatic hypermutation is due to intrinsic B cell defects, extrinsic exposure differences, or both.

Four insulin-binding BCRs (expressed by *n* = 19 cells) were identified in the clonally expanded Stage 1 T1D group only; these BCRs were isolated from two Stage 1 T1D individuals, both of whom were insulin autoantibody positive at the time of blood draw. It is possible that some of the non-insulin-binding, clonally expanded BCRs identified here (some of which came from MIAA-negative individuals) recognize other islet autoantigens, which we did not test. Three of the four Stage 1 T1D insulin-binding mAbs were identified in atypical-like memory cluster 3, with the fourth mAb originating from memory cluster 7. These insulin-binding BCRs were minimally mutated, non-class-switched, and exhibited avidity-driven insulin recognition. Nicholas et al. similarly isolated minimally mutated, low-affinity islet reactive B cells from autoantibody positive individuals and found that a majority of the clonally expanded, islet-reactive B cells were found in the autoantibody positive and T1D groups, with many of these clonally expanded cells residing in the atypical B cell, memory and plasmablast compartments.^17^ Findings from Nicholas et al. described low affinity, islet reactive B cells as polyreactive (defined by positivity for multiple islet autoantigens).^17^ In contrast, the insulin-binding B cells we isolated from the memory pool here did not exhibit polyreactivity to nuclear and cytoplasmic self-antigens by HEp-2 immunofluorescence. Additional studies will be required to address correlations between insulin-binding B cell polyreactivity, affinity, and epitope specificity.

Of note, insulin-binding BCRs that arose naturally in NOD mice have shown a similar non-inhibitable/avidity-based binding profile to insulin.^56^ This contrasts with mouse anti-insulin mAb125, modified here to include a human Fc for use as a positive control, which showed inhibitable binding to insulin, as in previous studies of the fully murine (original) version of this mAb.^43^ Anti-insulin mAb125 was derived from a mouse immunized with foreign (human) insulin, and thus did not originate from a true autoimmune response. The affinity of mAb125 was estimated to be 3 × 10^8^ M^−1^ for human insulin, but only 8 × 10^6^ M^−1^ for rodent insulin using ELISA-based methods.^43^ Despite this low affinity for (rodent) insulin self-antigen, mAb125 supports diabetes in NOD mice when it is expressed as a BCR transgene, showcasing the pro-pathogenic potential of low-affinity anti-insulin BCRs.^6^^,^^8^^,^^52^

Study limitations include the following. Four non-diabetic healthy control individuals were collected outside of TrialNet and were not screened for islet autoantibodies. The healthy control group was composed of non-diabetic, first-degree relatives of T1D individuals, which increases risk of T1D onset anywhere from 1.3% to 9%, as reviewed by Redondo et al.^65^ These statistics suggest it is unlikely that healthy control individuals represented in this cohort were destined to eventually develop diabetes; this is further supported by the numerous gene expression changes observed here between healthy controls and Stage 1 T1D individuals. We however cannot exclude the possibility that some of the healthy controls might have gone on to develop diabetes. Our power to identify gene expression differences and clonal expansion was limited by the small sample size and number of sequenced cells, particularly within less abundant B cell subsets like plasmablasts, and when comparing the insulin-binding and non-insulin binding B cells. Most of the anti-insulin BCRs identified here were from a single individual and do not capture T1D disease heterogeneity; rather, they contribute to the limited number of published human anti-insulin BCR sequences to begin to elucidate autoreactive B cell features in human T1D.

To our knowledge, this is the only ELISA-validated group of transcriptionally profiled human insulin-binding B cells isolated exclusively from Stage 1 T1D individuals. Our data identify upregulated genes involved in BCR membrane organization, signaling and antigen presentation in several B cell subsets isolated from Stage 1 T1D individuals compared to healthy controls. Future studies will address the functional consequences of these gene expression changes, with a goal of identifying new immunotherapy targets in T1D. Among Stage 1 T1D individuals, we identified an atypical-like memory cluster that exhibits impaired somatic hypermutation, minimal class switching, and contains insulin-binding B cell clones. The transcriptional profile of insulin-binding B cells appears distinct from non-insulin binding B cells. Technologies such as LIBRA-seq require pre-defined autoantigens and large blood volumes for identification of antigen-specific B cells.^16^ In contrast, the approaches outlined here combine immune repertoire, transcriptional, and phenotypic features through multi-omic profiling to enable identification of rare, autoantigen-binding B cells in small blood volumes and could be repurposed to identify B cells reactive against novel autoantigens (with antigen specificity determined via subsequent testing). These methods also hold potential for enabling study of autoreactive B cells changes with T1D disease progression.

## Supplementary Material

vlaf053_Supplementary_Data

## Data Availability

Raw sequencing data are available on the Sequence Read Archive under BioProject accession number PRJNA1252169. Sequences for monoclonal antibodies characterized in this study have been deposited to GenBank under accession numbers PX226776 - PX226841, listed in Table S4.
