# Supplementary material for: Stage 1 type 1 diabetes memory B lymphocytes transcriptionally differ from healthy controls and harbor insulin-binding specificities
Source: Immunohorizons. 2025 Nov 19;9(11):vlaf053. doi: 10.1093/immhor/vlaf053 (PMC12629600; doi:10.1093/immhor/vlaf053)
Supplement: vlaf053_Supplementary_Data [file vlaf053_supplementary_data.zip › Supplementary Information_IH_final.pdf]

Supplementary Material

Supplemental Table S1. TrialNet Pathway to Prevention Inclusion/Exclusion Criteria	
Inclusion Criteria	Exclusion Criteria
Have not been diagnosed with T1D	Diagnosis of diabetes
Between the ages of 2 and 45 and have a first-degree relative previously diagnosed with T1D	Previous or current use of medications to control hyperglycemia
Between the ages of 2 and 20 and have a second-degree relative previously diagnosed with T1D	Current use of immunosuppressive or immunomodulatory therapies

Supplemental Table S2. Overrepresented gene pathways in memory B cells in Stage 1 T1D individuals compared to healthy controls. Upregulated genes with fold change increase >1.2 and adjusted p value < 0.05 were included in the g:Profiler analysis.

GO.ID	Description	p value	Genes
GO:0048306	calcium-dependent protein binding	0.000798	S100A10,ANXA2,S100A11,PLSCR3,VAMP2,ANXA6,CALM2
GO:1905686	positive regulation of plasma membrane repair	0.000818	S100A10,ANXA2,AHNAK
GO:0003779	actin binding	0.00181	GSN,TAGLN2,PDLIM1,SPTAN1,COTL1,ARPC4,ANXA6,DBNL,ARPC1B,WASF2,PARVG,LSP1,MTSS1
GO:0071800	podosome assembly	0.00434	GSN,HCK,BIN2,DBNL
GO:1903729	regulation of plasma membrane organization	0.00818	GSN,S100A10,ANXA2,AHNAK
GO:1905684	regulation of plasma membrane repair	0.0161	S100A10,ANXA2,AHNAK
GO:0006091	generation of precursor metabolites and energy	0.0180	UCP2,COX5B,MTLN,LDHA,ETFB,TKT,CXXC5,NDUFA7,ATP5F1C,NDUFS5,RUBCNL,NDUFA3,ATP5MF
GO:0008092	cytoskeletal protein binding	0.0218	GSN,SPACA9,TAGLN2,TUBGCP2,PDLIM1,SPTAN1,BLOC1S2,COTL1,ARPC4,ANXA6,TLN1,CALM2,CALM1,DBNL,ARPC1B,WASF2,PARVG,LSP1,MTSS1
GO:0015986	proton motive force-driven ATP synthesis	0.0391	NDUFA7,ATP5F1C,NDUFS5,NDUFA3,ATP5MF,ATP5MC3
GO:0019855	calcium channel inhibitor activity	0.0402	MCUB,CALM2,CALM1
GO:0045296	cadherin binding	0.0481	RPS26,TAGLN2,ANXA2,S100A11,PDLIM1,AHNAK,SPTAN1,LDHA,TLN1,DBNL,WASF2

Supplemental Table S3. CITE-seq antibody panel

Format	Target:	Clone	Cat #	Company
TotSeq C	CD20	2H7	302363	BioLegend
TotSeq C	CD21	Bu32	354923	BioLegend
TotSeq C	CD27	O323	302853	BioLegend
TotSeq C	CD38	HIT2	303543	BioLegend
TotSeq C	C0251 antihuman hashtag 1	LNH-94/2M2	394661	BioLegend
TotSeq C	C0251 antihuman hashtag 2	LNH-94/2M2	394663	BioLegend
TotSeq C	C0251 antihuman hashtag 5	LNH-94/2M2	394669	BioLegend

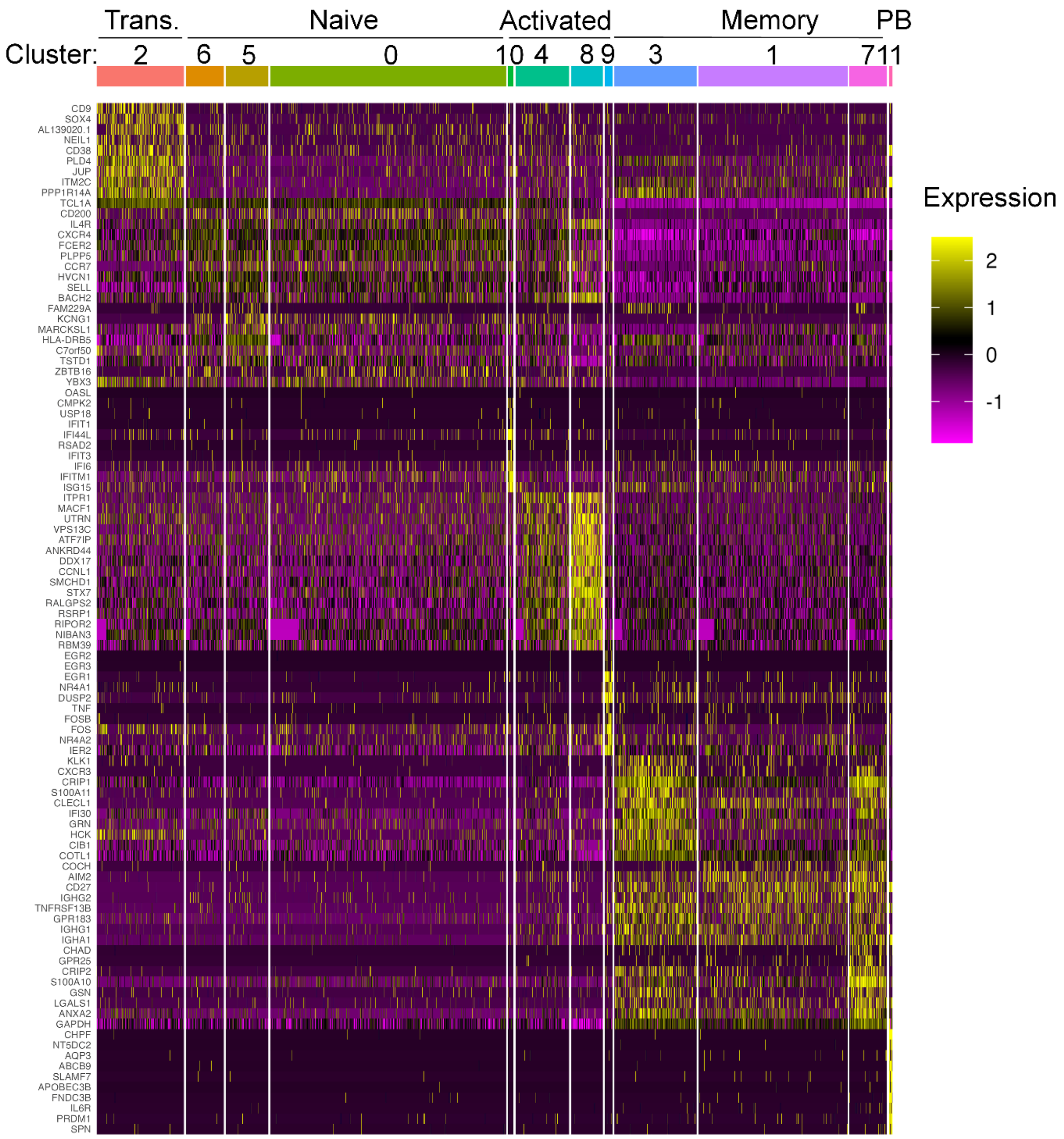

Supplemental Figure S1. Distinct gene expression profiles define B cell clusters. Purified $CD19^+$ $CD3^-$ cells isolated from n = 9 Stage 1 T1D individuals and n = 8 healthy controls were profiled using single-cell RNA-seq technology as in Figure 1 and Methods. The top 10 differentially expressed genes by cluster are shown on the heatmap.

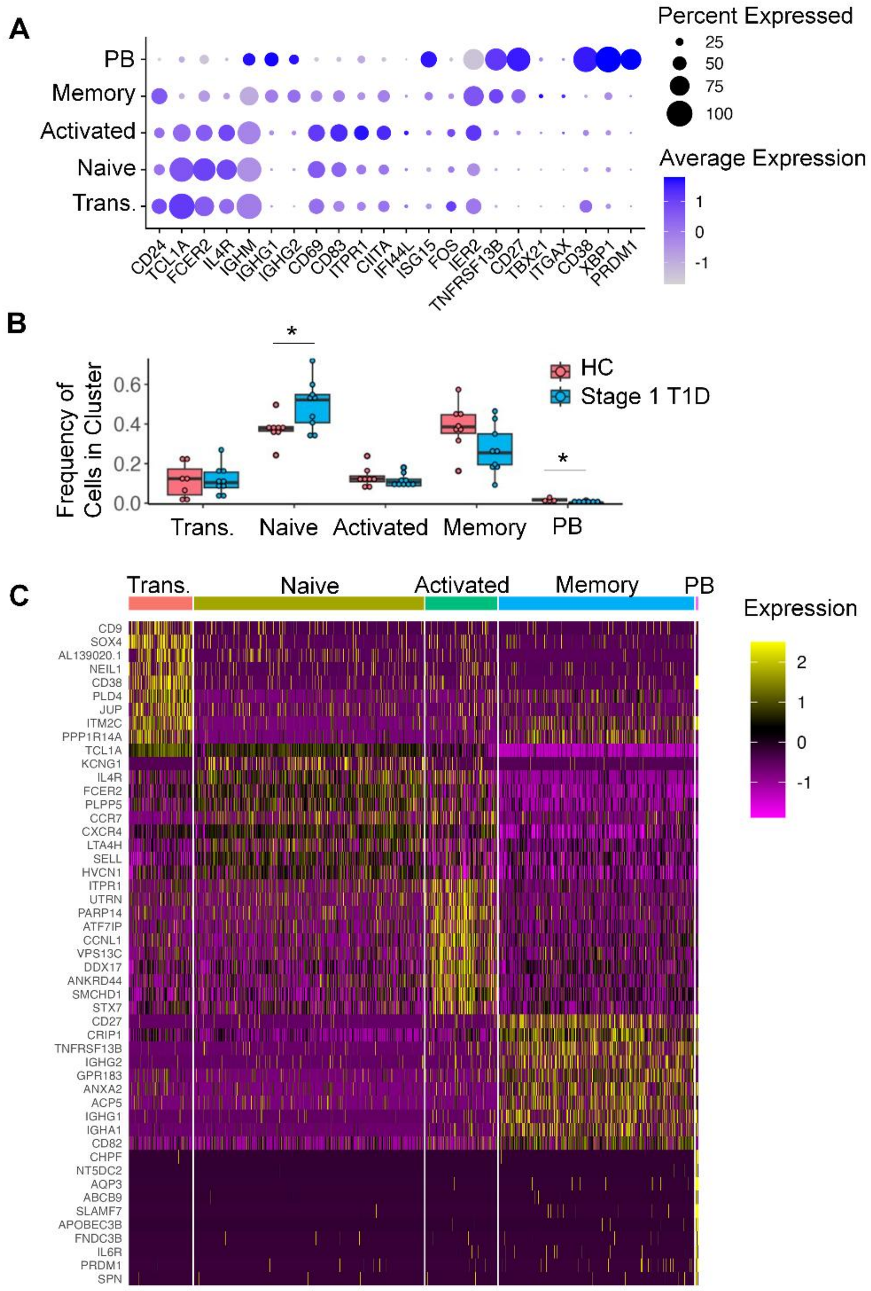

Supplemental Figure S2. B cell clusters were collapsed into transitional, naïve, activated, memory and plasmablast subsets. B cell clusters across all individuals were identified as in Methods and Figure 1, which were manually assigned to the indicated B cell subsets. **[A]** Dot plot of manually selected gene expression profiles used to assign major B cell subsets. **[B]** Boxplots show the mean frequency of each B cell cluster by disease group, individual donors are plotted as points. A wilcoxon rank sum test was used for all pairwise group comparisons of the frequency of cells per subset (healthy vs. Stage 1 T1D), ***** $p < 0.05$, otherwise groups comparisons were NS. **[C]** The top 10 differentially expressed genes by subset are shown on the heatmap.

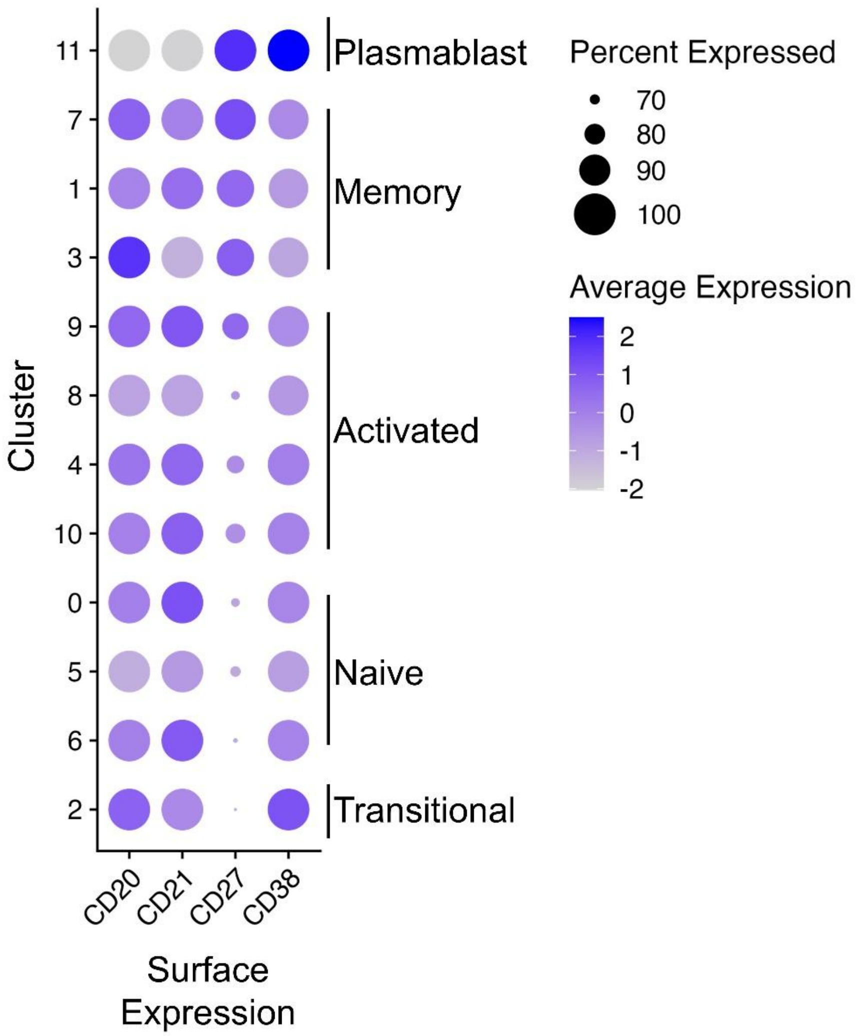

Supplemental Figure S3. Surface expression of key B cell phenotypic markers aligns with transcriptionally defined B cell clusters. B cell clusters were identified as in Methods and Figure 1 and manually assigned to B cell subsets. Dot plot shows CD20, CD21, CD27, and CD38 surface expression in each transcriptionally-defined cluster as determined by CITE-seq.

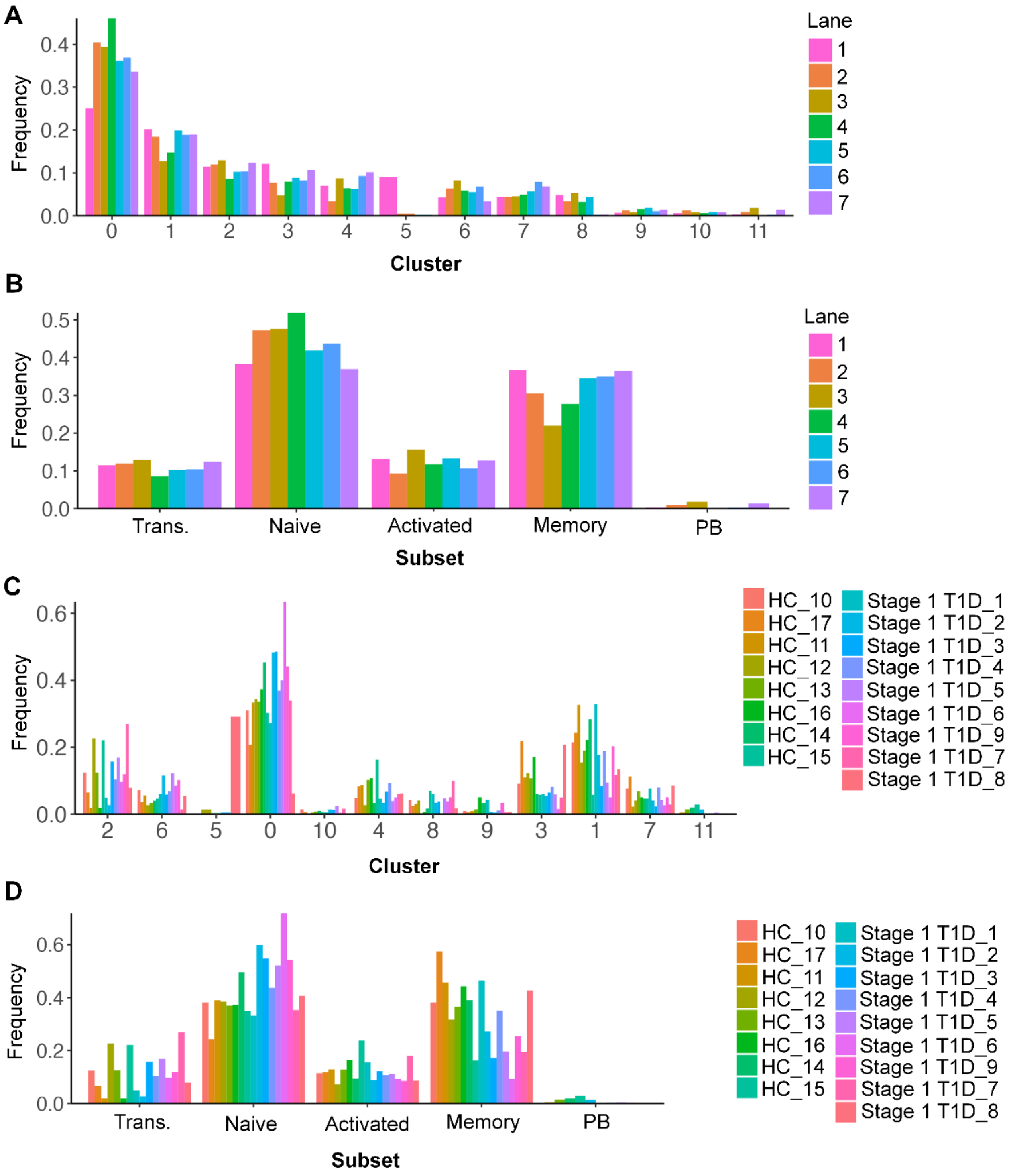

Supplemental Figure S4. An individual donor or lane did not drive transcriptional B cell clustering. Single-cell profiling of $CD19^{+}$ $CD3^{-}$ cells was performed as in Methods. Frequency of cells in each lane (for 10X Genomics profiling) is plotted across **[A]** cluster and **[B]** subset. Frequency of cells from each individual is plotted across **[C]** cluster and **[D]** subset.

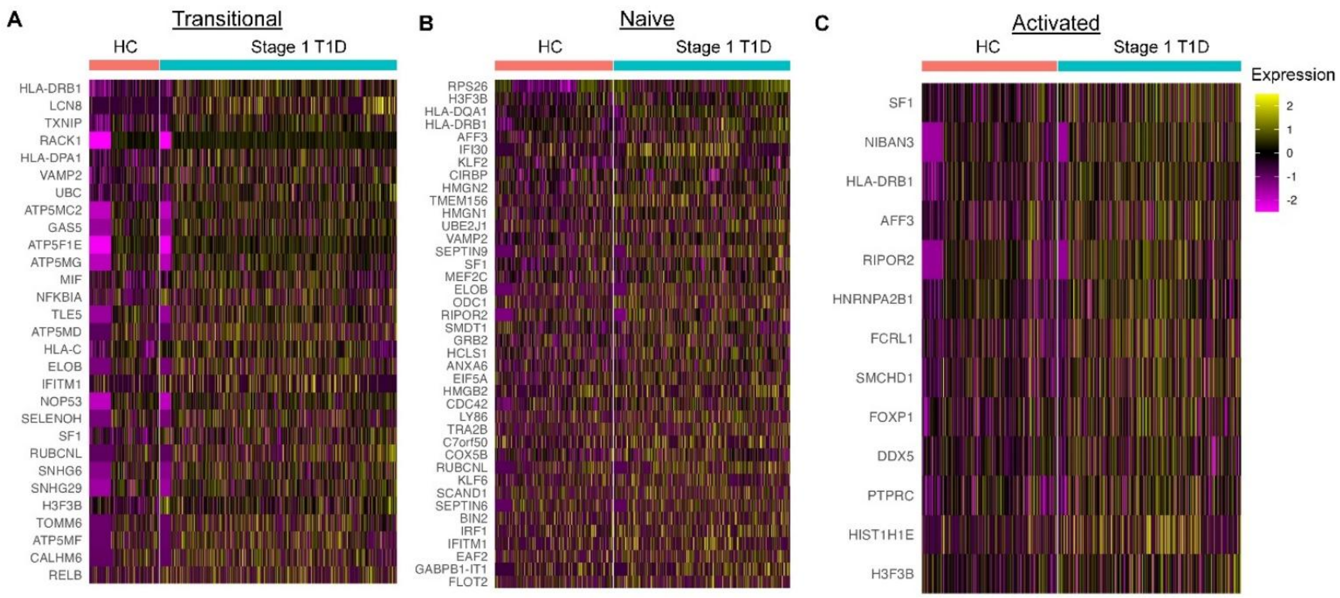

Supplemental Figure S5. Differential gene expression in Stage 1 T1D individuals compared to healthy controls in the transitional, naïve, and activated B cell compartments. $CD19^+$ $CD3^-$ cells were single-cell profiled as in Methods. The top 40 genes upregulated in Stage 1 T1D B cells compared to healthy controls in the **[A]** transitional, **[B]** naïve and **[C]** activated subsets are shown in the heatmaps. The following cutoff criteria were required for inclusion: fold change >1.2, adjusted p value < 0.05, and expressed in > 30% of cells in either disease group. No differentially expressed genes (DEGs) met the cutoff in the plasmablast subset, which is omitted here.

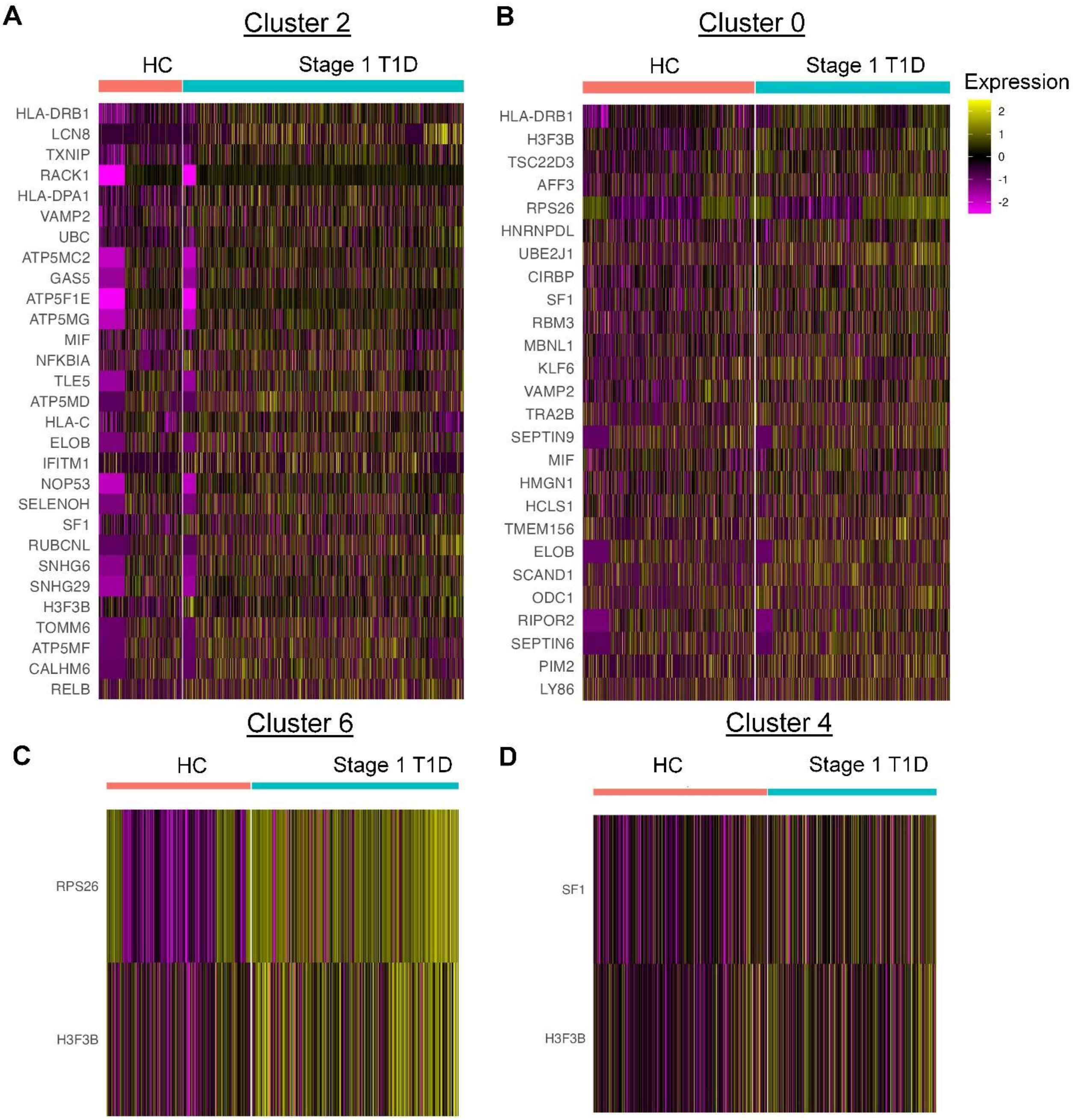

Supplemental Figure S6. Differential gene expression in Stage 1 T1D individuals compared to healthy controls in non-memory clusters. The top 40 genes upregulated in Stage 1 T1D B cells compared to healthy controls (profiled as in Methods) in **[A]** transitional cluster 2, **[B]** naïve cluster 0, **[C]** naïve cluster 6, and **[D]** activated cluster 4 are shown in the heatmaps. The following cutoff criteria were required for inclusion: fold change >1.2, adjusted p value < 0.05, and expressed in > 30% of cells in either disease group. No DEGs met the cutoff in clusters 5, 8, 9, 10, and 11, which are omitted here.

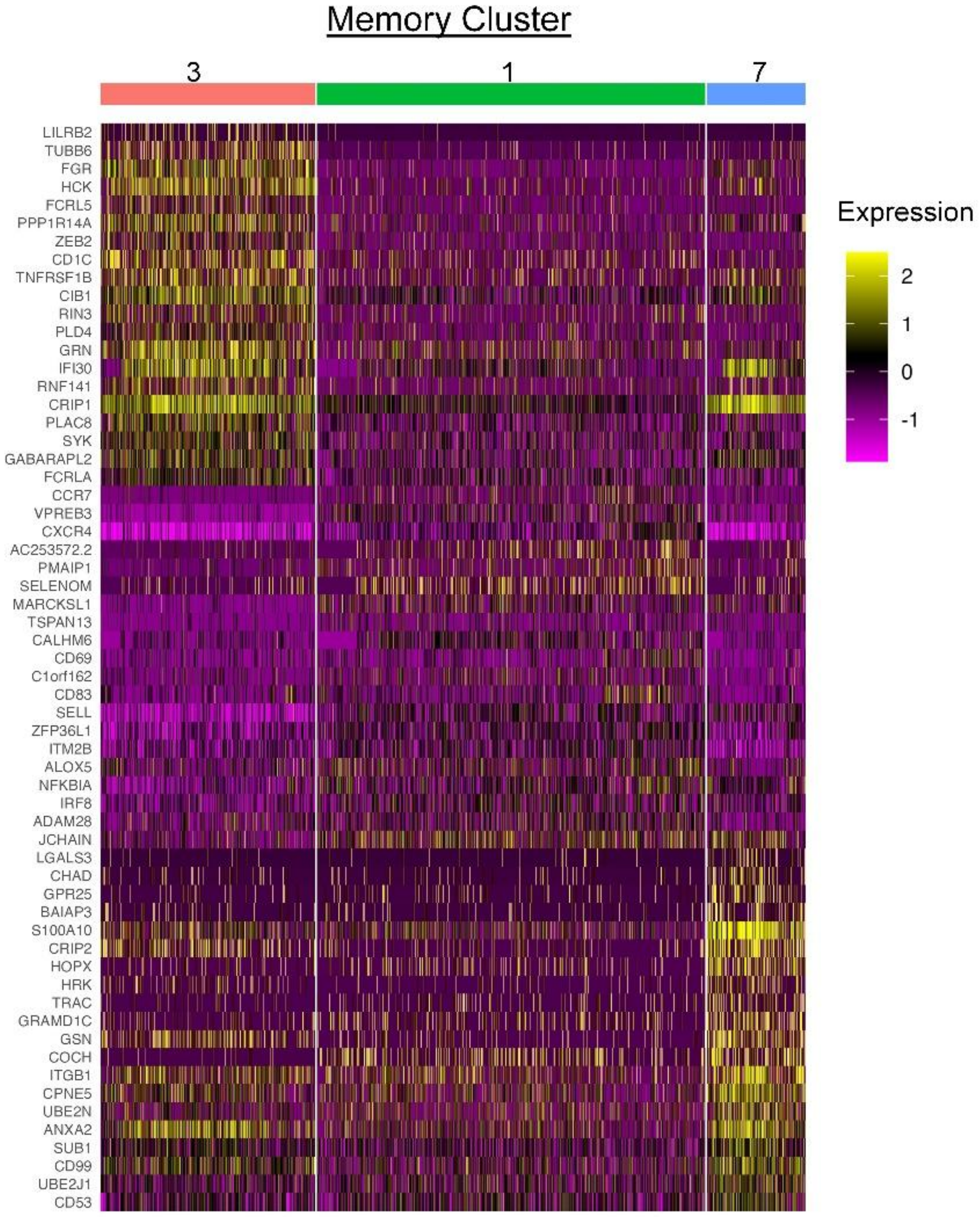

Supplemental Figure S7. Memory clusters exhibit transcriptional heterogeneity. Single-cell profiling was performed on $CD19^{+}$ $CD3^{-}$ cells as in Methods and memory clusters were identified as in Figure 1. The top 20 differentially expressed genes by memory cluster across all donors are shown on the heatmap.

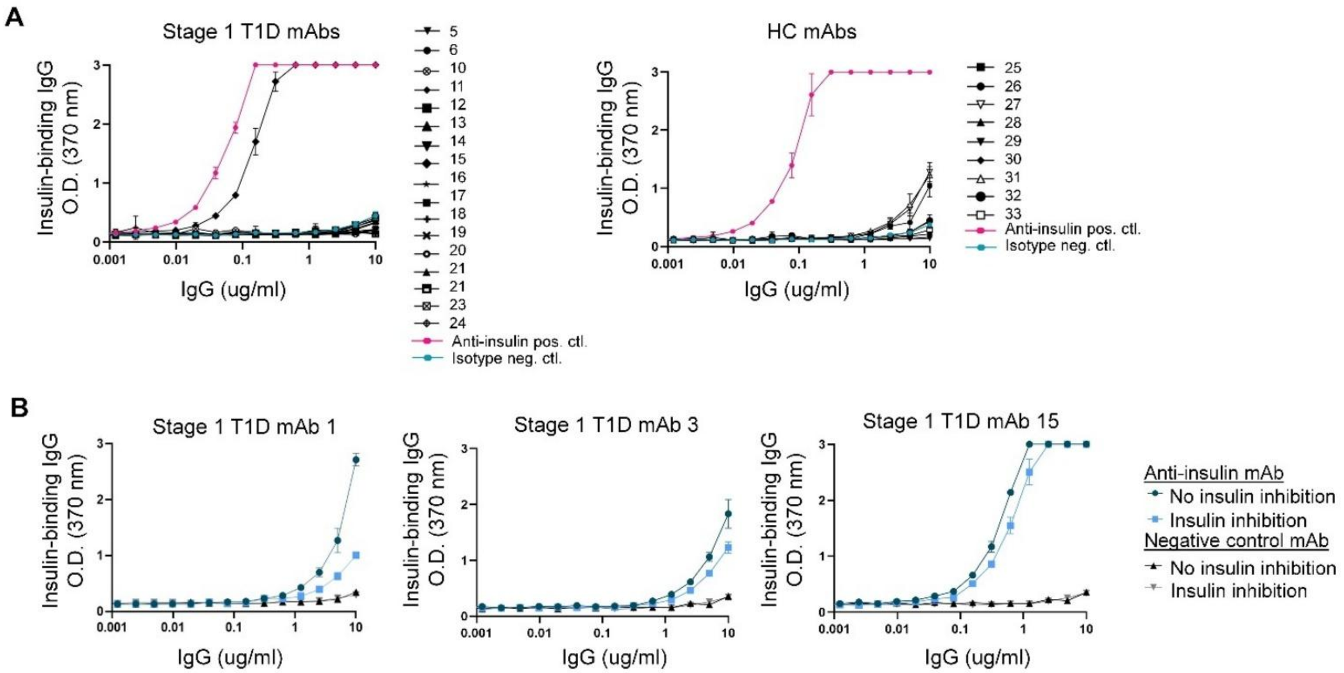

Supplemental Figure S8. Insulin binding and inhibition curves for clonally expanded BCRs. Clonally expanded BCRs were identified, expressed as mAb, and screened for insulin binding as in Figure 6 and Methods. **[A]** Insulin binding ELISA curves for clonally expanded BCRs, Stage 1 T1D BCRs (n=2 clones per clonotype, left) and HC BCRs (n ≥ 2 clones per clonotype, right). **[B]** For BCRs that met the positive cutoff for insulin binding in Figure 6, percent inhibition of insulin binding with the addition of soluble insulin was determined, as in Figure 7 and Methods. Insulin ELISA binding curves of the candidate insulin-binding mAbs and the negative isotype control are shown here.

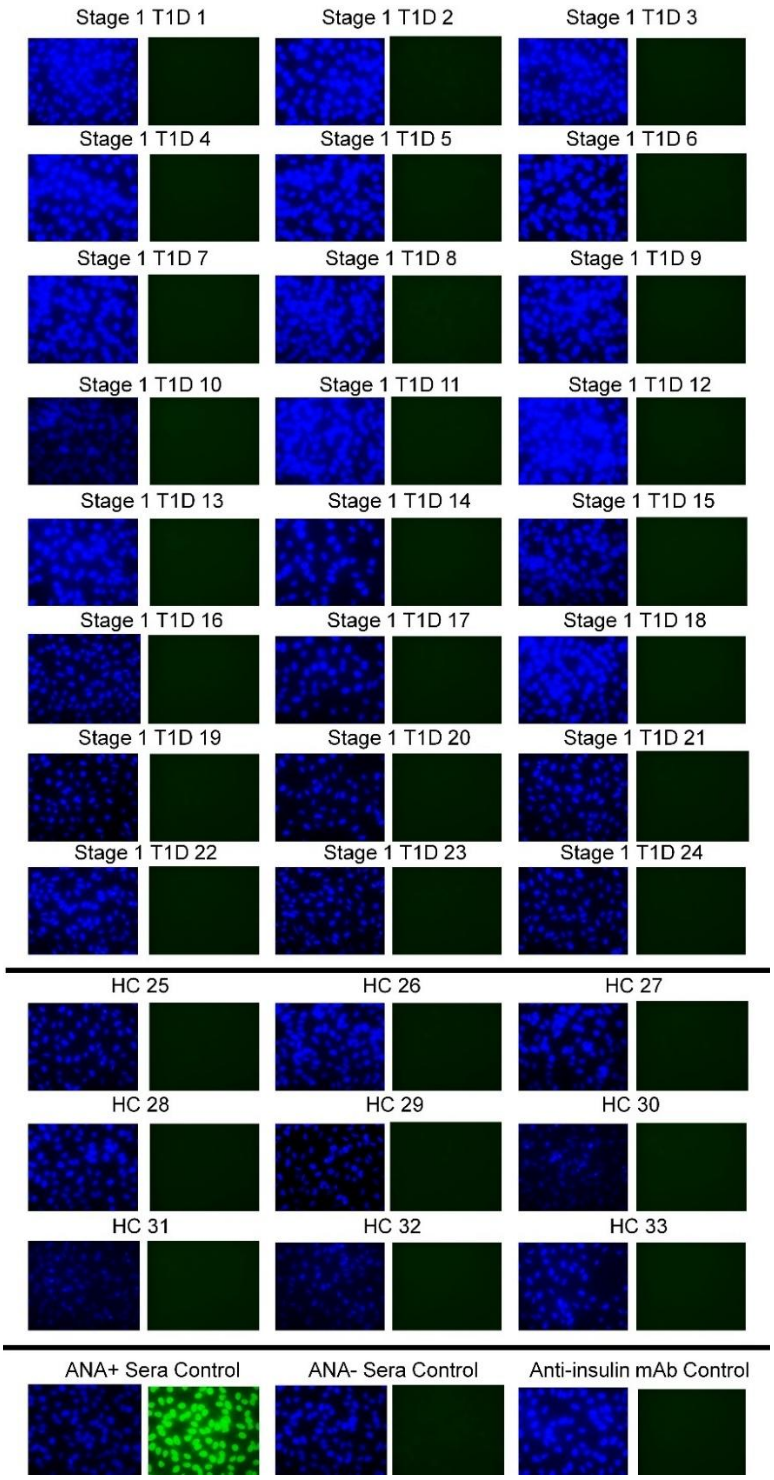

Supplemental Figure S9. HEp-2 autoreactivity was not observed in clonally expanded BCRs expressed as mAbs that were isolated from Stage 1 T1D or healthy control individuals. HEp-2 reactivity measured for the clonally expanded Stage 1 T1D and healthy control BCRs expressed as mAbs, as identified in Figure 7 and Methods. DAPI (blue) stained the nucleus. FITC (green) stained the HEp-2 autoantigen reactive mAbs.
